# Ribosomal stress and Tp53-mediated neuronal apoptosis in response to capsid protein of the Zika virus

**DOI:** 10.1038/s41598-017-16952-8

**Published:** 2017-11-30

**Authors:** Lukasz P. Slomnicki, Dong-Hoon Chung, Austin Parker, Taylor Hermann, Nolan L. Boyd, Michal Hetman

**Affiliations:** 1 Kentucky Spinal Cord Injury Research Center and the Department of Neurological Surgery, University of Louisville, Louisville, Kentucky 40292 USA; 2 Pharmacology & Toxicology, University of Louisville, Louisville, Kentucky 40292 USA; 3 Center of Predictive Medicine and the Department of Microbiology & Immunology, University of Louisville, Louisville, Kentucky 40292 USA; 4 Cardiovascular Innovation Institute and the Department of Physiology, University of Louisville, Louisville, Kentucky 40292 USA

## Abstract

We report here that in rat and human neuroprogenitor cells as well as rat embryonic cortical neurons Zika virus (ZIKV) infection leads to ribosomal stress that is characterized by structural disruption of the nucleolus. The anti-nucleolar effects were most pronounced in postmitotic neurons. Moreover, in the latter system, nucleolar presence of ZIKV capsid protein (ZIKV-C) was associated with ribosomal stress and apoptosis. Deletion of 22 C-terminal residues of ZIKV-C prevented nucleolar localization, ribosomal stress and apoptosis. Consistent with a casual relationship between ZIKV-C-induced ribosomal stress and apoptosis, ZIKV-C-overexpressing neurons were protected by loss-of-function manipulations targeting the ribosomal stress effector Tp53 or knockdown of the ribosomal stress mediator RPL11. Finally, capsid protein of Dengue virus, but not West Nile virus, induced ribosomal stress and apoptosis. Thus, anti-nucleolar and pro-apoptotic effects of protein C are flavivirus-species specific. In the case of ZIKV, capsid protein-mediated ribosomal stress may contribute to neuronal death, neurodevelopmental disruption and microcephaly.

## Introduction

Zika virus (ZIKV) is a mosquito-transmitted RNA virus that belongs to the virus family *Flaviviridae* and causes usually asymptomatic infection in humans^1^. However, a causative link has been established between prenatal exposure to ZIKV and severe congenital microcephaly^1^. ZIKV ability to disturb neurodevelopment is consistent with a neurotropic potential of other arthropod-transmitted flaviviruses^1^. For instance, mosquito-transmitted West Nile virus (WNV) and Japanese encephalitis virus (JEV) infect CNS neurons causing neurological diseases which may include neuronal loss and permanent deficits in brain function^2^. However, ZIKV appears to be a uniquely potent brain teratogen^1^. Such effect is likely due to its ability to penetrate the placenta and infect fetal brain^3^. In contrast, innate anti-viral response may prevent ZIKV from reaching the brain in adults.

Congenital microcephaly is caused by insufficiency of neurogenesis that may originate from (*i*) depletion of neuroprogenitor cells (NPCs) due to their apoptosis and/or pre-mature differentiation, (*ii*) inhibition of NPC proliferation, and, (*iii*) apoptosis of newly generated neurons^4–6^. ZIKV has a high potential to infect human or rodent NPCs and to a lesser extent developing neurons^7–11^. ZIKV reduces NPC proliferation, induces their premature differentiation and activates apoptosis of NPCs and immature neurons. However, a question remains as to what are the mechanisms behind such cytotoxic effects of ZIKV.

Recent work has suggested that ZIKV protein NS4A and, to lesser extent, NS4B may impair NP proliferation by inhibiting the growth promoting AKT pathway^12^. In addition, Tp53 has been proposed to mediate ZIKV-induced apoptosis but its ZIKV-related activation mechanism remains obscure^13^. Thus, experiments are needed to determine which ZIKV proteins induce cell death of the developing brain cells and what the mechanisms behind their anti-survival effects in that cellular context are.

Although flaviviruses replicate on the ER membrane^14,15^, at least a fraction of some flaviviral proteins is found in the nucleus of the infected cells, including nucleolar presence of WNV-, JEV-, or Dengue virus (DENV) capsid protein (protein C)^16^. Being structural components of flaviviruses, capsid proteins are very abundant in the infected cells^17^. They interact with multiple host proteins including ribosomal biogenesis factors (RBFs)^18–21^. Some of these interactions may be toxic to the infected cells. For instance, immature form of WNV-C was shown to cause nucleolar sequestration of the Tp53 inhibitor MDM2/HDM2 leading to Tp53-mediated apoptosis in a human cell line^22^.

The nucleolus, where flaviviral capsid proteins are often found, is a center of ribosomal biogenesis^23^. Dysregulation of ribosomal biogenesis triggers ribosomal stress (RS)^24^. In most cases, such a response relies on interactions between the Tp53 ubiquitin ligase MDM2/HDM2 and ribosomal components that are no longer incorporated into ribosomes^24^. Specifically, RPL11, RPL5, and 5S rRNA, acting as a complex (the 5S ribonucleoprotein particle /5S RNP/), bind and inhibit MDM2, stabilizing Tp53 and producing cell cycle arrest and/or apoptosis^25,26^. When RNA-Polymerase-1 (Pol1), which initiates ribosomal biogenesis, is inhibited, nucleolar structure is disrupted^23^. Thus, loss of the granular component (GC) of the nucleolus including nucleoplasmic dispersion of its prominent protein components such as nucleophosmin-1 (NPM1/B23) or PES1 provides a convenient way of monitoring this type of RS^27^. However, the RS-Tp53 pathway may also be activated without structural disruption of the nucleolus and with normal- or increased activity of Pol1^27,28^.

Several lines of evidence suggest that RS may be involved in pathogenesis of neurodevelopmental diseases. First, RS activates RPL11/Tp53-dependent apoptosis of immature cortical neurons^29^. Second, acephalic mouse fetuses were produced when Pol1 was inhibited selectively in Nestin-positive NPCs^30^. Similarly, just a single injection of the anti-ribosomal drug 5-fluorouracil to pregnant rats induces microcephaly of the offspring^31^. Finally, recent description of the nucleolar proteome from the developing rat brain included 9 proteins whose human counterparts are mutated in microcephaly syndromes^32^. Several of these proteins are expected to participate in brain ribosomal biogenesis as verified experimentally for a newly identified RBF, LARP7^32^. Hence, RS may contribute to neuroteratogenic effects of various mutations, toxins and infectious agents by inhibiting neurogenesis and/or activating apoptosis of immature neurons. Therefore, the current study was initiated to examine role of RS in ZIKV-mediated damage of the developing brain.

## Results

### Nucleolar disruption in ZIKV-infected neural cells

Rat NPCs (rNPCs) were infected with two different strains of ZIKV including the African strain MR766 and the Puerto Rico strain PRVABC59 (PRV). Neurosphere formation was disrupted by either strain of ZIKV as early as at 2 days post infection (dpi, Supplementary Fig. S1). It was accompanied by cell attachment to the plates and disappearance of neurospheres suggesting NPC differentiation (Supplementary Fig. S1). These effects were associated with moderate infection of rNPCs as, at least, at 4 dpi, only 8.3–10% cells were positive for flaviviral E protein (Flavi-E) dependent on ZIKV strain (Supplementary Fig. S1). In addition, increased apoptosis was observed in MR766-infected rNPCs (Supplementary Fig. S1). Hence, our findings are consistent with reports of disrupted neurogenesis in ZIKV-infected human NPC (hNPC) neurosphere cultures and suggest that the virus perturbs growth and/or maintenance of mammalian NPCs^9,33^.

**Figure 1:**
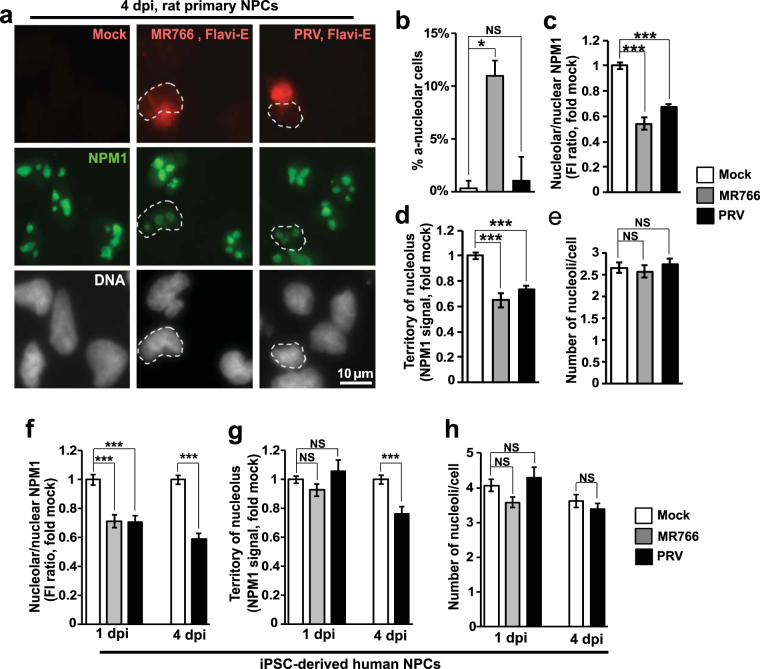
Nucleolar stress in ZIKV-infected neuroprogenitor cells. Freshly isolated rat embryonic NPCs (rNPCs) or monolayer cultures of human iPSC-derived NPCs (hNPCs) were infected with ZIKV strains MR766 or PRVABC59 at MOI 0.1. The rNPCs were grown as neurospheres for 3 days post infection (dpi), dispersed and cultured as a monolayer for 16 h to enable microscopic analysis at a single cell level; the hNPCs were maintained in a monolayer culture. After fixation, co-immunofluorescence staining was performed for the ZIKV infection marker (the flaviviral E protein, Flavi-E) and the nucleolar marker nucleophosmin-1 (NPM1/B23); nuclear DNA was counterstained with Hoechst-33258. Additional NPC data on ZIKV infection and cytotoxicity are presented in Supplemenary Figs S1 and S2. **(a)** Representative images depicting non-apoptotic ZIKV-infected rNPCs (*i*.*e*. lacking apoptotic chromatin condensation). Dotted lines mark nuclear contours of these cells; note reduced fluorescence intensity (FI) of NPM1. **(b)** At least MR766 infection increased fraction of non-apoptotic cells without NMP1-positive nucleoli. **(c,d)** Quantification of NPM1 signal confirmed ZIKV-induced reduction of fluorescence intensity (FI) as well as nucleolar territory. **(e)** Nucleolar number was unaffected by ZIKV. **(f)** Nucleolar stress in ZIKV-infected hNPCs as revealed by reduced nucleolar FI of NPM1 signal at dpi 1 (representative images are shown in Supplementary Fig. S2). **(g)** At dpi 4, there was also a significant reduction in NPM1-defined nucleolar territory of PRV-infected cells. **(h)** Nucleolar number was unaffected. At dpi 4, cells that survived MR766 infection showed similar anti-nucleolar effects as those infected with PRV (Fig. S3). Immunofluorescence staining for an additional marker of the nucleolar GC, PES1 confirmed negative effects of ZIKV infection on hNPC nucleoli (Supplementary Fig. S4). Data represent two independent experiments including 2 sister cultures/experiment in **(b)**, and, at least 58 **(c**–**e)** or 27 **(f**–**h)** randomly selected individual cells with no signs of chromatin condensation that were analyzed for each condition; error bars are SEM. Data were analyzed by *u*-test **(b)** or one-way ANOVA and Tukey’s post-hoc tests **(c-h)**, NS, p > 0.05; *p < 0.05; ***p < 0.001.

To monitor effects of ZIKV on structural integrity of the nucleolus co-immunofluorescence staining for Flavi-E and the nucleolar marker NPM1 was performed in ZIKV-infected rNPCs. In mock-infected rNPCs most NPM1 signal was concentrated in nucleoli with just 0.36% ± 0.36% cells with uncondensed nuclei that did not contain at least one NPM1-positive nucleolus (Fig. 1a-b). In nucleoli of the ZIKV-infected (*i*.*e*. Flavi-E-positive) cells but not the uninfected neighbor cells nucleolar NPM1 signal was reduced (Fig. 1a–d). First, infection with MR766 increased fraction of rNPCs without any nucleolar NPM1 signal (11% ± 0.72%, Fig. 1b); PRV had no significant effects on that parameter (Fig. 1b). Second, in cells with nucleolar NPM1 staining, each strain of ZIKV reduced the nucleoplasm-normalized nucleolar NPM1 signal by about 40% (Fig. 1c). Reductions of the NPM1-defined nucleolar territory but not number of nucleoli/cell were also observed (Fig. 1d,e). Importantly, as nucleolar morphology analysis was limited to cells with uncondensed chromatin, mitotic and/or apoptotic re-organization of the chromatin did not cause such anti-nucleolar effects of ZIKV.

ZIKV-induced nucleolar abnormalities were also present in human induced pluripotent stem cell-derived NPCs (hNPCs). In this system, MR766 or PRVABC59 led to relatively rapid declines of cell viability with at least 75% reductions at 3- or 5 dpi, respectively (Supplementary Fig. S2). Infection rates were also higher than in rat NPCs (maximum fractions of infected cells of 84% or 49% with the MR766 or PRV at 2- or 4 dpi, respectively, Supplementary Fig. S2). At 1 dpi, when Flavi-E signal was detected in 11% or 0.25% cells that were infected with MR766 or PRV, respectively, each ZIKV strain reduced nucleolar NPM1 signal intensity by nearly 30% (Supplementary Fig. S2 and Fig. 1f). Importantly, at that time hNPC viability was unaffected by ZIKV (Supplementary Fig. S2). Similar reduction of NPM1 signal intensity and additional nucleolar shrinkage were present in surviving cells that were infected with PRV for 4 days (Fig. 1f,g). Nucleolar number was not significantly altered by ZIKV at any time (Fig. 1h). In addition, fraction of cells with absence of nucleolar NPM1 did not change after ZIKV infection except a slight increase from 0 to 3.2% in MR766-infected hNPCs at 4 dpi. At that time, MR766-infecetd cells which contained NPM1-positive nucleoli displayed similar reductions of nucleolar NPM1 as at 1 dpi (Supplementary Fig. S3). Finally, in ZIKV-infected hNPCs, anti-nucleolar effects were also evident when nucleolar GC was visualized by immunostaining with a human-specific antibody against the ribosomal biogenesis factor PES1 (Supplementary Fig. S4). These findings suggest that in ZIKV-infected NPCs, structural integrity of the nucleolus is disturbed.

**Figure 2:**
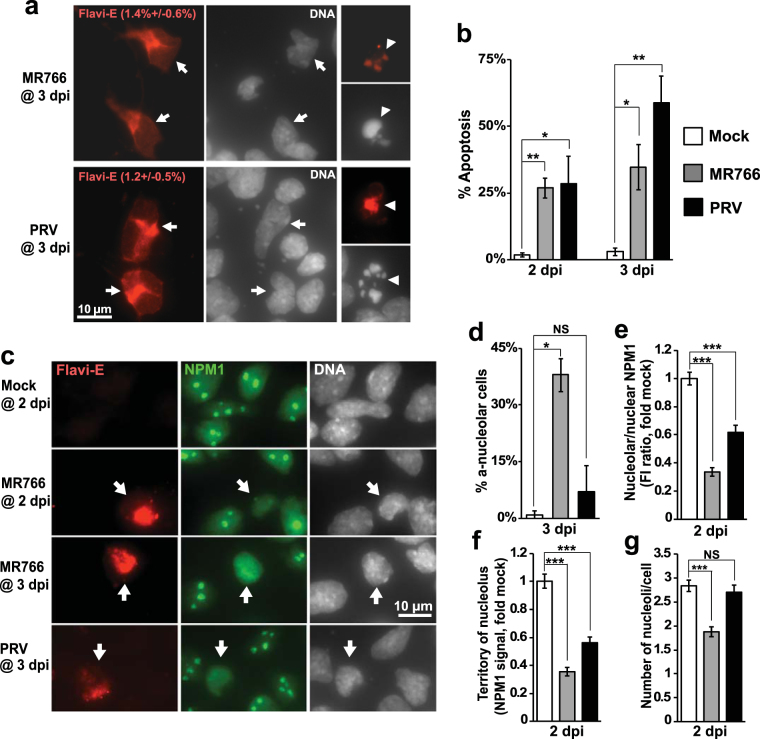
Pro-apoptotic and anti-nucleolar effects of ZIKV in rat embryonic cortical neurons. At day *in vitro* (DIV) 1, neurons were infected with ZIKV strains MR766 or PRVABC59 (PRV) at MOI 0.1. **(a)** Representative images of infected neurons that were identified by Flavi-E immunofluorescence; apoptotic condensation of nuclear chromatin was visualized by counterstaining DNA with Hoechst-33258. Arrows identify infected neurons with healthy nuclei; arrowheads indicate infected neurons with signs of apoptosis (condensation and/or fragmentation of chromatin). **(b)** Increased apoptosis of ZIKV-infected neurons. **(c)** Representative images depicting non-apoptotic, ZIKV-infected neurons (arrows) that were co-immunostained for the nucleolar marker NPM1. Note reduced fluorescence intensity (FI) of the nucleolar NPM1 signal, apparent shrinkage of NPM1-positive nucleolar territory and increased nucleoplasmic signal of NPM1 indicative of its release from nucleoli. **(d)** ZIKV MR766 infection increased fraction of non-apoptotic cells without NMP1-positive nucleoli. **(e**,**f)** Quantification of nucleolar NPM1 signal confirmed ZIKV-induced reduction of its intensity as well as territory. **(g)** Nucleolar number was reduced by MR766 but not PRV. In **(b**,**d)** data represent 6 sister cultures from three independent experiments; in **(e**–**g)**, images of at least 60 randomly selected individual cells with no signs of apoptotic chromatin condensation were analyzed for each condition; such images were collected from two independent experiments; error bars are SEM. Data were analyzed by *u*-test **(b**,**d)** or one-way ANOVA and Tukey’s post-hoc tests **(e**–**g)**, NS, p > 0.05; *p < 0.05; ***p < 0.001.

Pathological evidence as well as data from mouse models indicate that postmitotic cortical neurons may become infected with ZIKV and are highly sensitive to ZIKV-induced apoptosis^3,11,34^. Therefore anti-nucleolar effects of ZIKV infection were evaluated in embryonic cortical neurons. Consistent with previously published cell culture data^7,35^, neuronal infection rate was relatively low with 1.2 or 1.4% cells being Flavi-E-positive at 3 dpi with PRV or MR766, respectively (Fig. 2a). However, infection with either strain of ZIKV was associated with increased neuronal apoptosis (Fig. 2a,b).

Co-staining for Flavi-E, NPM1, and, DNA revealed that in many Flavi-E-positive- but non-apoptotic neurons both nucleolar NPM1 intensity and size of the nucleolus appeared to be reduced (Fig. 2c). Infection with MR766- but not PRV increased fraction of a-nucleolar cells with most NPM1 translocated to the nucleoplasm and lack of a clearly identifiable nucleolus (Fig. 2c,d). Moreover, in MR766- or PRV-infected neurons that maintained nucleolar enrichment of NPM1, the nucleus-normalized fluorescence intensity of nucleolar NPM1 as well as NPM1-defined nucleolar territory was reduced (Fig. 2e,f). However, MR766 effects on these parameters were greater than those of PRV (MR766 vs. PRV, p < 0.001 or p < 0.01 for nucleolar NPM1 intensity or territory, respectively, Tukey’s *post-hoc* test; Fig. 2e,f). Also, number of nucleoli per nucleus declined in response to MR766 but not PRV (Fig. 2g).

Combined analysis of nucleolar disruption data from all three cell types revealed that host cell was a significant factor in determining severity of RS (Table 1, Supplementary Table S1). Specifically, in MR766-infected rat neurons all nucleolar status parameters were reduced to a greater extent than in rNPCs (Table 1). In case of PRV, reductions in two parameters were enhanced in neurons while two others were similar in both cell types (Table 1). Thus, in postmitotic embryonic neurons, ZIKV infection results in nucleolar disruption which appears to be more pronounced than in NPCs.

**Table 1:** After infection with ZIKV, nucleolar disruption is stronger in rat embryonic cortical neurons than in rat embryonic cortical NPCs.

Virus Strain	MR766				PRVABC59			
State of the Nucleolus (No)	anucleolar cells (%)	NMP1 FI in No (fold control)	Territory of No (NPM1, fold control)	No number (fold control)	anucleolar cells (%)	NMP1 FI in No (fold control)	Territory of No (NPM1, fold control)	No number (fold control)
Values in Neurons (2 dpi, ±SEM)	19.53 ± 3.22	0.3357 ± 0.0295	0.3569 ± 0.0305	0.6621 ± 0.0366	7.46 ± 0.712	0.6156 ± 0.0514	0.562 ± 0.0426	0.9534 ± 0.0507
Values in NPCs (4 dpi, ±SEM))	11 ± 0.72	0.5427 ± 0.0459	0.6506 ± 0.0562	0.9172 ± 0.0538	1.12 ± 1.12	0.6729 ± 0.0266	0.7302 ± 0.0347	0.9781 ± 0.047
Neurons vs. NPCs	p < 0.05^*MW*^	p < 0.001^*lsd*^	p < 0.001^*lsd*^	p < 0.001^*lsd*^	p < 0.05^*MW*^	p > 0.05^*lsd*^	p < 0.01^*lsd*^	p > 0.05^*lsd*^

^*MW*^Mann-Whitney *u*-test; ^*lsd*^ two-way ANOVA and Fisher’s LSD *post hoc* test (ANOVA F values/factors: ZIKV infection, host cell type/are presented in Supplementary Table S1).

### Nucleolar localization of the ZIKV capsid protein (ZIKV-C) in neural cells

**Figure 3:**
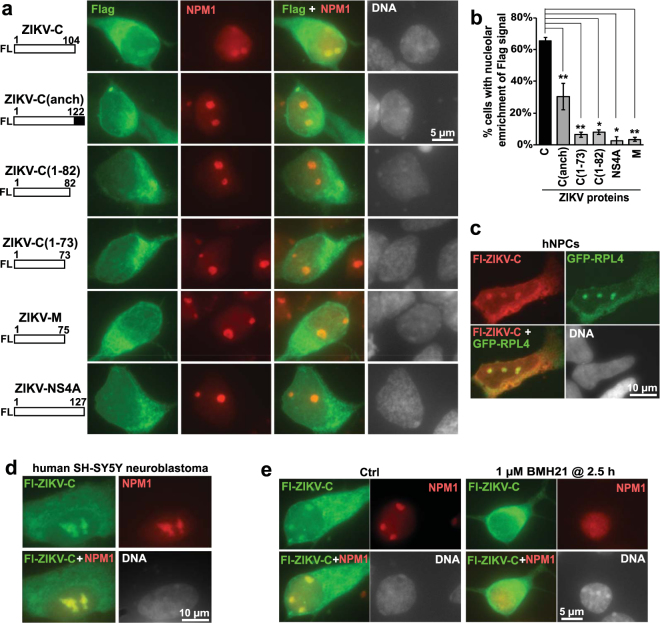
Nucleolar localization of the ZIKV capsid protein ZIKV-C. **(a)** DIV2 rat embryonic cortical neurons were transfected for 48 h with expression vectors for Flag (Fl)-tagged ZIKV-C, its variants, or, other ZIKV proteins as indicated (0.15 μg plasmid DNA/3.5*10^5^ cells); to avoid apoptosis due to possible ZIKV-C-mediated ribosomal stress, an expression plasmid for a dominant negative mutant of Tp53 was also added (Tp53-DD, 0.15 μg plasmid DNA/3.5*10^5^ cells). Representative images of co-immunofluorescence for Flag and the nucleolar marker NPM1 revealed strong perikaryal expression of all ZIKV proteins including predominantly cytosolic localization and nucleolar enrichment of a fraction of Fl-ZIKV-C in many but not all Fl-positive cells (more images of Fl-ZIKV-C immunofluorescence are presented in Supplementary Fig. S6. **(b)** Quantification of nucleolar enrichment of Fl-ZIKV proteins. To equalize apparent differences in expression efficiency between Fl-ZIKV-C and other constructs, a β-gal expression plasmid was added to the transfections to provide a consistent transfection marker (150 ng plasmid DNA/3.5*10^5^ cells, all other components as described for panel **(a)**, see text for more details). Nucleolar enrichment analysis was performed in β-gal/Fl-double-positive cells. Nucleolar enrichment was observed for Fl-ZIKV-C and, to lesser extent, for the immature, membrane anchored version of Fl-ZIKV-C (C/anch/). Nucleolar enrichment was rare for other constructs including C-terminal deletion mutants of ZIKV-C (C/1-73/ and C/1-82/). Data represent averages of 6 sister cultures from three independent experiments; NS, p > 0.05; *p < 0.05; **p < 0.01 (*u*-test). **(c**,**d)** hNPCs or SH-SY5Y cells were transfected with Fl-ZIKV-C (150 ng plasmid DNA/10^5^ cells) and its localization was analyzed by Fl immunofluorescence 48 h later; nucleolar enrichment was confirmed by co-transfection of a nucleolar/ribosomal marker GFP-RPL4 (hNPCs, 150 ng plasmid DNA/10^5^ cells) or co-immunostaining for NPM1 (SH-SY5Y cells). **(e)** Neurons were transfected as in (**a**), and treated with a Pol1-specific inhibitor, BMH21 as indicated. Nucleolar enrichment of both NPM1 and Fl-ZIKV-C was disrupted by BMH21 (nucleolar enrichment of Fl-ZIKV-C was present in 50.6 ± 0.6% or 15.8 ± 5.3% control- or BMH21-treated cells, respectively as determined in two independent experiments).

As capsid proteins of several non-ZIKV flaviviruses have a high potential to interact with host RBFs and localize to the nucleoli^16,18–21^, anti-nucleolar effects of ZIKV may be caused by ZIKV-C. To evaluate such a possibility, Flag (Fl)-tagged ZIKV-C was overexpressed in otherwise intact rat embryonic neurons. In these studies, a synthetic ZIKV-C cDNA gene was used to produce ZIKV-C with amino acid sequence identical to that of three ZIKV strains with direct links to microcephaly; it differs by 1 or 3 amino acid residues from PRV or MR766, respectively (Supplementary Fig. S5). The dominant-negative mutant of Tp53 (DN-Tp53) was also co-expressed to protect cells from a potential RS and RS-mediated apoptosis that may have been expected in response to ZIKV-C. Most of the mature Fl-ZIKV-C (ZIKV-C) was present in the perikaryal cytoplasm (*i*.*e*. cell soma cytoplasm, Fig. 3a and Supplementary Fig. S6). However, in many cells a fraction of Fl-ZIKV-C was also found in nuclei including enrichment in NPM1-positive nucleoli (Fig. 3a and Supplementary Fig. S6). Mostly cytosolic signal was observed for other variants of ZIKV-C including the immature, membrane anchored ZIKV-C (ZIKV-C/anch/) and C-terminal deletion mutants (ZIKV-C/1–73/ and ZIKV-C/1–82/) that lacked the C-terminal basic RNA binding domain and a portion of the α-helical domain 4 (α4, Fig. 3a). Almost exclusively cytosolic signal was found in case of two other ZIKV proteins (NS4A and M, Fig. 3a).

However, Fl-ZIKV-C-positive cells were 5 times more frequent than those expressing other variants of C or non-C ZIKV proteins; in most Fl-ZIKV-C-transfected cells expression levels of Fl signal were relatively low. To exclude that such expression disparities affected apparent differences in nucleolar localization, neurons were co-transfected with the expression plasmids for Fl-ZIKV proteins and β-gal. Frequency of cells that were double positive for Fl and β-gal as well as intensity of Fl signal in such cells was similar regardless of Fl-ZIKV construct that was expressed. Therefore, nucleolar enrichment of the Fl signal was determined in β-gal-positive cells. In such a population, 64% or 32% cells displayed nucleolar enrichment of Fl-ZIKV-C or Fl-ZIKV-C(anch), respectively (Fig. 3b). Reduced nucleolar enrichment of ZIKV-C(anch) suggests that membrane trapping lowers ability of ZIKV-C to accumulate in nucleoli. For ZIKV-C deletion mutants, nucleolar enrichment was present in less than 10% cells suggesting that the basic C-terminal RNA-binding domain and/or the α4 helical domain plays a role in the nucleolar localization of ZIKV-C^17^. Such localization appears to be specific for ZIKV-C as neither ZIKV-M nor ZIKV-NS4A accumulated in nucleoli. Nucleolar enrichment of Fl-ZIKV-C was also observed in hNPCs or the human neuroblastoma cell line SH-SY5Y (Fig. 3c,d).

Interestingly, pharmacological inhibition of Pol1 with the Pol1-specific drug BMH-21 reduced nucleolar enrichment of either Fl-ZIKV-C or NPM1 (Fig. 3e). Dependence of nucleolar enrichment of ZIKV-C on ongoing rRNA transcription suggests that nucleolar localization of ZIKV-C may require binding to rRNA or rRNA-interacting nucleolar proteins such as NPM1^36^.

As currently there are no available antibodies against mature ZIKV-C it is unclear whether in ZIKV-infected neural cells, ZIKV-C is nucleolar. However, such localization is expected based on data from non-neuronal cells that were infected with related flaviviruses including JEV or DENV^37,38^.

### Nucleolar disruption by the overexpressed ZIKV-C

**Figure 4:**
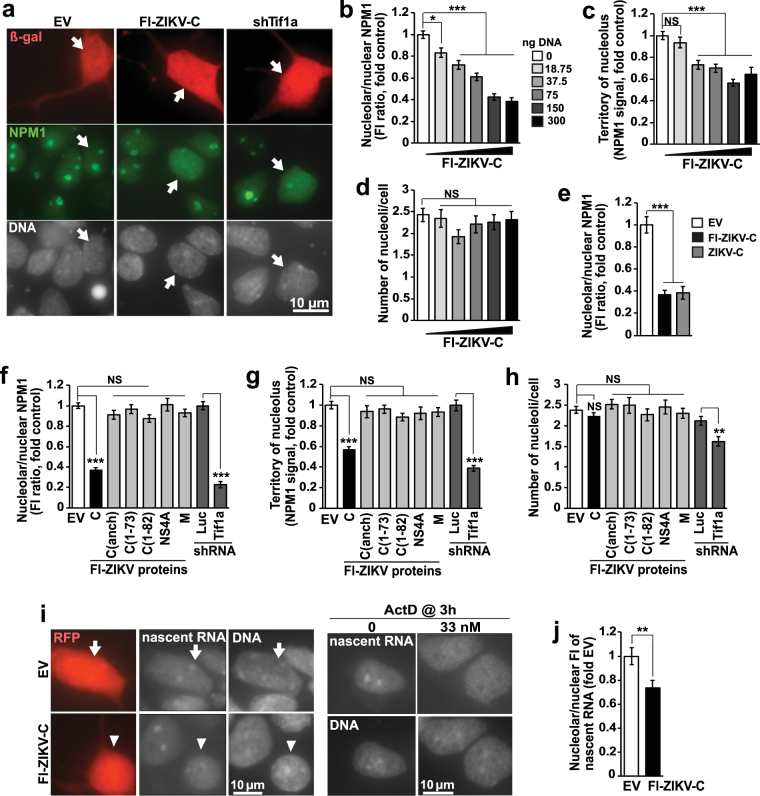
Disruption of neuronal nucleoli by the overexpressed ZIKV-C. Transfections of ZIKV proteins were as in Fig. 3b with β-gal or empty cloning vector (EV, pBact-16-pl) used as a transfection marker or a negative control, respectively; additional controls included shRNAs targeting Renilla luciferase (shLuc) or the Pol1 co-activator Tif1a (shTif1a, used as a positive control for RS); all analyses were performed 48 h after transfection. **(a)** Representative images of transfected (*i*.*e*. β-gal-positive neurons, arrows) that were co-immunostained for NPM1; 150 ng plasmid DNA of EV or ZIKV-C/3.5*10^5^ cells or 300 ng plasmid DNA of shTif1a/3.5*10^5^ cells were used. Fl-ZIKV-C or shTif1a reduced NPM1 signal in the nucleolus while increasing it in the nucleoplasm (more images are in Supplementary Fig. S7). **(b**,**c)** Plasmid DNA dose-dependent reduction of intensity- and territory of the nucleolar NPM1 signal (one-way ANOVA, factor DNA dose, p < 0.001). **(d)** Fl-ZIKV-C did not affect nucleolar number. **(e)** Similar reductions of NPM1 signal in the nucleolus after transfections of Fl-ZIKV-C or untagged ZIKV-C. **(f**–**h)** Plasmid dosage was as in **(a)**. Nucleolar NPM1 signal was affected by ZIKV-C or shTif1a but not the membrane bound precursor form ZIKV-C(anch) or C-terminal deletion mutants of mature ZIKV-C or other ZIKV proteins **(f**,**g)**; nucleolar number was reduced only by shTif1a **(h)**. **(i**–**j)** *In situ* run on assay revealed reduction of nascent RNA signal in nucleoli of ZIKV-C-transfected neurons suggesting lower activity of Pol1. Two days after transfections (as in **(a)**), cells were incubated with 5-ethynyluridine (5-EU) for 2 h, fixed and 5-EU-labelled nascent RNA was detected using Click-It chemistry. Then, β-gal immunofluorescence was performed to identify transfected cells (arrows). In nucleoli of Fl-ZIKV-C-transfected neurons, whole nucleus-normalized accumulation of nascent RNA was moderately reduced **(j)**; however, Pol1 inhibition may be potentially underestimated as average whole nucleus signal was also reduced (Supplementary Table S2). At a low concentration of 33 nM, ActD abolished all nascent RNA signal in nucleoli validating its specificity. Data represent averages of at least 39 cells/condition from two- **(b**–**e**,**j)** or three independent experiments **(f**–**h)**; NS, p > 0.05; *p < 0.05; ***p < 0.001 (one-way ANOVA and Tukey’s *post-hoc* tests).

As compared to cells that were transfected with an empty cloning vector, cells receiving increasing doses of Fl-ZIKV-C plasmid DNA displayed progressive reductions in nucleolar NPM1 signal intensity as well as NPM1-defined nucleolar territory (Fig. 4a–c and Supplementary Fig. S7). However, complete loss of nucleolar NPM1 was not observed; average number of NPM1-positive nucleoli per cell was unaffected either (Fig. 4d). Conversely, NPM1 signal in the nucleoplasm was increased (Fig. 4a,b). When untagged ZIKV-C was overexpressed, similar reductions in nucleolar NPM1 staining were observed as with Fl-ZIKV-C (Fig. 4e).

These anti-nucleolar effects of ZIKV-C overexpression were similar to those after knockdown of the Pol1 co-activator TIF1A whose depletion is a well established stimulus for pro-apoptotic RS in neurons (Fig. 4a and f–h)^29^. No significant effects on nucleolar morphology were observed after overexpression of ZIKV-C variants with reduced ability to reside in the nucleolus (Fig. 4f–h). Moreover anti-nucleolar effects of ZIKV-C were specific as neither ZIKV-NS4A nor ZIKV-M affected neuronal nucleoli (Fig. 4f–h). Finally, *in situ* run on assay revealed that such effects were associated with reduced nucleolar accumulation of nascent RNA suggesting lower activity of Pol1 (Fig. 4i,j, Supplementary Table S2). Although nucleolar accumulation of nascent RNA was visible in similar fractions of ZIKV-C- or empty vector-transfected cells (64.6- or 67.4%, respectively), relative intensity of the nucleolar signal was down by 27% (Fig. 4i,j, Supplementary Table S2). Therefore, at least in post-mitotic neurons overexpression of ZIKV-C is sufficient to perturb nucleolar structure and reduce ribosomal biogenesis.

### Neuronal apoptosis in response to overexpressed ZIKV-C is mediated by the ribosomal stress pathway

**Figure 5:**
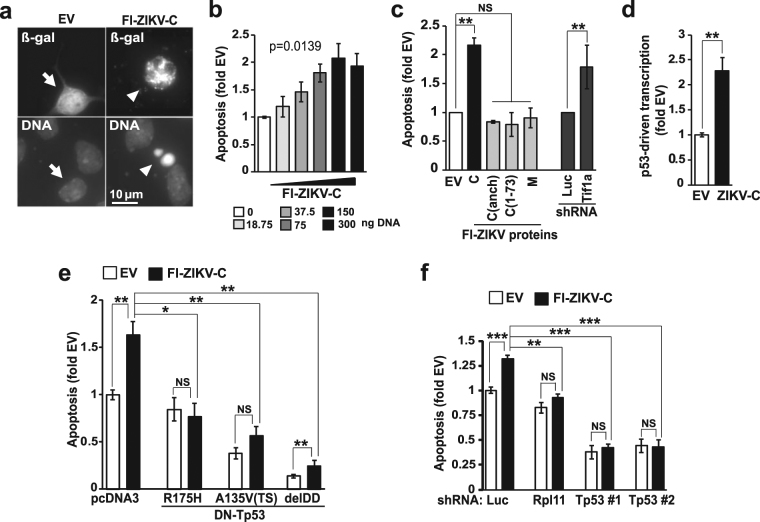
Ribosomal stress-mediated apoptosis of ZIKV-C-overexpressing neurons. DIV2 neurons were transfected as described for Fig. 4 except DN-Tp53 constructs were added only as indicated in **(e)** and omitted from all other transfections. Unless indicated otherwise, ZIKV protein expression constructs were used at 150 ng plasmid DNA/3.5*10^5^ cells. In **(d)** besides Fl-ZIKV-C or its EV control, transfections included luciferase reporter constructs (p53-driven firefly luciferase and EF1α-driven Renilla luciferase, 0.1 ng plasmid DNA/10^5^ cells, each; luciferase assay was performed at 24 h post transfection). All analyses except luciferase assays were at 48 h post transfection. **(a)** Representative images of transfected (*i*.*e*. β-gal-positive) neurons. Counterstaining with the DNA dye Hoechst-33258 revealed condensation and fragmentation of nuclear chromatin in ZIKV-C-transfected neurons (arrowheads), a non-apoptotic neuron is pointed by arrows. **(b)** ZIKV-C induces apoptosis in a plasmid DNA-dose dependent manner (Kruskal-Wallis ANOVA, factor DNA dose, p < 0.05). **(c)** Similar apoptotic response to ZIKV-C or shTif1a but not ZIKV-C(anch) or ZIKV-C(1–73) or ZIKV-M. **(d)** ZIKV-C increased Tp53-driven transcription as determined by activity of a co-transfected p53-driven firefly luciferase reporter plasmid. **(e**,**f)** As expected for ribosomal stress-mediated apoptosis that involves the RPL11-Tp53 pathway, ZIKV-C-transfected neurons were protected by co-transfection of DN-Tp53 variants, or previously validated shRNAs against Rpl11^27^, or rat Tp53^39^. The shTp53 plasmids also reduced activity of the Tp53-driven luciferase reporter in either vehicle- or nutlin-treated neurons confirming their ability to inhibit Tp53 in that system (Supplementary Fig. S8). Note that as compared to a control cDNA expression vector (pcDNA3.1, **(e)**) control shRNA (shLuc, **(f)**), increased baseline apoptosis in non-ZIKV-C-transfected neurons (16.6% or 28.3%, respectively, p < 0.05, *u*-test). Due to such baseline increases, ZIKV-C was relatively less pro-apoptotic in neurons that received shLuc than pcDNA3.1 (1.3- vs. 1.6 fold, **(f)** vs. **(e)**). Data represent averages ± SEM of 4 **(b)** or 6 **(d)** sister cultures from two independent experiments, or 6 sister cultures from three independent experiments **(c**,**e**,**f)**; NS, p > 0.05; *p < 0.05; **p < 0.01 (*u*-test).

In neurons that were transfected with Fl-ZIKV-C but not DN-Tp53 (Tp53-DD) a plasmid DNA dose-dependent increase of apoptosis was observed (Fig. 5a,b). No apoptotic response was observed with other variants of ZIKV-C including C(anch) and C(1–73) as well as ZIKV-M (Fig. 5c). Finally, pro-apoptotic effects of ZIKV-C were similar to those after knockdown of the Pol1 co-activator, TIF1A (Fig. 5c). Thus, in case of ZIKV-C there is a good correlation between nucleolar localization, disruption of nucleolar NPM1 and ability to induce neuronal apoptosis.

As ribosomal stress-induced neuronal apoptosis involves pro-apoptotic activity of Tp53 and its ribosomal stress-specific regulator, RPL11^27^ their role in neurotoxicity of ZIKV-C was investigated. Overexpression of Fl-ZIKV-C stimulated Tp53-driven transcription as demonstrated using a Tp53-driven luciferase reporter construct (Tp53-Luc, Fig. 5d). Importantly, ability of the Tp53-Luc to detect Tp53 activity was confirmed using previously validated shRNAs against rat Tp53^39^. In neurons, such shRNAs reduced reporter activity under basal conditions or after treatment with the Tp53 activator nutlin (Supplementary Fig. S8).

When Fl-ZIKV-C was co-expressed with several dominant negative mutants of Tp53 (DN-Tp53) ZIKV-C-induced apoptosis was reduced (Fig. 5e). While such protective effects suggest a role of Tp53 in ZIKV-C-induced apoptosis, one should note that many DN-Tp53 mutants may have gain of function activities beyond inhibition of wild type Tp53 (WT-Tp53) including cross inhibition of Tp53 relatives such as p63 or p73 and modulation of several other transcriptional regulators^40^. However, such gain of function effects are often mutant-specific^40^. Hence, similar inhibition of ZIKV-C-induced apoptosis by each of the three different DN-Tp53s that were tested here (two missense mutations: R174H /human/ and V135A/mouse/ and a miniprotein Tp53-DD that lacks amino acid residues 15–301 of mouse WT-Tp53) suggests that their protective effects were mediated by inhibition of WT-Tp53. Indeed, shRNAs against the RS-specific Tp53 regulator RPL11 or Tp53 itself abolished apoptotic response to ZIKV-C (Fig. 5f).

### ZIKV-C-mediated disruption of the nucleolus is specific to post-mitotic neurons

Although in proliferating cells including hNPCs or SH-SY5Y a fraction of Fl-ZIKV-C was present in the nucleoli (Fig. 3c,d), its overexpression had only relatively limited effects on nucleolar NPM1 staining (Supplementary Fig. S9). Moreover, there were no significant effects on nucleolar nascent RNA accumulation or Tp53-driven transcription (Supplementary Fig. S9). Therefore, unlike in post-mitotic neurons, ZIKV-C overexpression appears to be insufficient to induce the RS-Tp53 pathway in proliferating cells.

### In neurons, DENV-C, but not WNV-C, disrupts nucleolus and induces apoptosis

**Figure 6:**
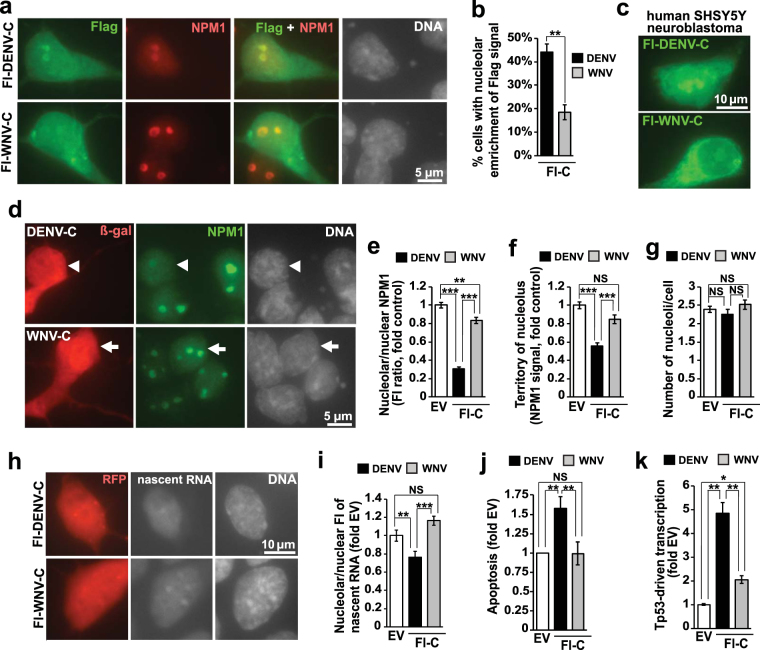
Capsid protein of Dengue virus (DENV-C) compromises nucleolar integrity and induces neuronal apoptosis. DIV2 neurons or SH-SY5Y cells were transfected with expression plasmids for Flag-(Fl) tagged DENV-C or WNV-C as described for Fig. 4 except DN-Tp53 was omitted from neuronal transfections in panels **(j)** and **(k)**. **(a**–**c)** In neurons and SH-SY5Y cells nucleolar enrichment appeared to be stronger for DENV-C than WNV-C including higher fraction of cells displaying such an enrichment **(b)**, and, higher fluorescence intensity (FI) in the nucleolus (FI quantifications in Supplementary Fig. S10). **(d**–**g)** In β-gal-positive neurons that were co-transfected with expression plasmids for β-gal and flaviviral Cs, DENV-C but not WNV-C reduced nucleoplasm-normalized NPM1 signal intensity in the nucleolus and NPM1-positive nucleolar territory (arrowheads in **(d)**, black bars in **(e**,**f)**); arrows point a Fl-WNV-C overexpressing neuron. However, number of NPM1-positive nucleoli was unaffected **(g)**. **(h**,**i)** *In situ* run on assay revealed that whole nucleus-normalized nucleolar accumulation of nascent RNA was reduced in DENV-C but not WNV-C-transfected neurons. In both cases, nascent RNA signal was also reduced in whole nuclei suggesting inhibition of extranucleolar transcription and/or lower uptake of 5-EU into cells (Supplementary Table S2). Hence, anti-Pol1 effects of DENV-C may be potentially underestimated. **(j)** Increased neuronal apoptosis in response to DENV-C- but not WNV-C. **(k)** Tp53 reporter assay was performed as for Fig. 5d. DENV-C and WNV-C activated Tp53-driven transcription; the activation was stronger with the RS-inducing DENV-C than WNV-C. Data represent averages ± SEM of 6- **(b**,**j)** or 9 sister cultures **(k)** from three independent experiments, or, at least 51 cells- from three independent experiments **(e**–**g**,**i)**; NS, p > 0.05; *p < 0.05; **p < 0.01, ***p < 0.001 (*u*-test in **(b**,**j**,**k)**; one-way ANOVA with Tukey’s *posthoc* tests in **(e**–**g**,**i)**).

As ZIKV-related flaviviruses including DENV and WNV have a neurotropic potential one could wonder if their capsid proteins may engage similar neurotoxic mechanisms as ZIKV-C^1,41,42^. Both DENV-C and WNV-C are highly basic and have similar domains as ZIKV-C with ZIKV-C sequence identity at 39.4% or 46.2%, respectively (Supplementary Fig. S5)^17^. Indeed, besides strong cytosolic presence, overexpressed Fl-tagged DENV-C or WNV-C were also observed in neuronal nucleoli (Fig. 6a). However, both frequency of such cells as well as relative nucleolar enrichment as compared to the whole nucleus was greater for DENV-C than for WNV-C (Fig. 6b and Supplementary Fig S10). Moreover, while WNV-C had only minor effects on nucleolar NPM1 signal intensity but not nucleolar NPM1 territory, DENV-C reduced those parameters at least as much as ZIKV-C (Figs 4a–g and 6d–f). However, there was no effect on number of nucleoli (Fig. 6g). In addition, DENV-C, but not WNV-C, reduced nucleolar accumulation of nascent RNA (Fig. 6h,i, Supplementary Table S2). The reduction was moderate (0.75 fold empty vector control) but significant and was reminiscent of that observed with ZIKV-C (compare Figs 6i to 4j). These anti-nucleolar effects were correlated with DENV-C-induced neuronal apoptosis (Fig. 6j). No increase in apoptosis was observed with WNV-C (Fig. 6j). Finally, overexpression of DENV-C or WNV-C increased Tp53-driven transcription to 4.8- or 2- fold of empty vector control (Fig. 6k). In WNV-C-transfected neurons, such a nucleolar disruption-unrelated activation of Tp53 may be due to direct inhibition of MDM2 by WNV-C^22^. However, it appears to be insufficient to activate neuronal apoptosis. Taken together, similarly to ZIKV-C, DENV-C, but not WNV-C, engages cytotoxic RS to induce apoptosis in postmitotic neurons. Hence, pro-RS potential of flaviviral capsid proteins appears to be virus species-specific and may be related to distinct interactions with host cell proteome.

## Discussion

Our studies have revealed (i) presence of RS including nucleolar disruption in brain cells that were infected with ZIKV, and, (ii) ability of the overexpressed capsid protein ZIKV-C to localize to the nucleoli, induce RS and activate RS-mediated neuronal apoptosis. Therefore, inhibition of ribosomal biogenesis is directly involved in neurocytopathic effects of ZIKV and may lead to neurodevelopmental defects that are observed after fetal infection with that pathogen.

Viral interactions with host cell nucleolus are observed with many RNA viruses including positive strand RNA viruses such as *Flaviviridae* ^43^. Thus, specific viral proteins including the capsid protein traffic through the nucleolus interacting with nucleolar proteins which may be recruited to support viral replication. For instance, NPM1, or, the RNA helicase DDX56, or, the RNA binding protein nucleolin bind to capsid proteins and contribute to viral particle production of JEV, WNV, or, DENV, respectively^19–21^. Consistent with such observations, mutations that target residues critical for viral protein localization to the nucleolus often compromise viral titers and give rise to attenuated viral strains as reported for JEV or the arterivirus porcine reproductive and respiratory syndrome virus (PRRSV)^37,44^. One could also expect that such a hijacking of cellular RBFs could perturb cellular ribosomal biogenesis and induce RS leading to RS-mediated cytopathic effects including cell cycle arrest and/or Tp53-mediated apoptosis^24^. However, to the best of our knowledge, there were no prior reports of RS contribution to cytopathic effects of any virus. Hence, the current manuscript provides the first piece of evidence for such a role of RS as exemplified in ZIKV-infected neurons.

Interestingly, possible negative effects on ribosomal biogenesis including ZIKV-like nucleolar disruption were reported for several neuropathogenic RNA viruses. For instance, the poliovirus 3Cpro protease inhibits Pol1 co-activators such as upstream binding factor (UBF) and selectivity factor-1 (SL1)^45^. Schmallenberg virus is a representative of *Orthobunyaviridae* which has documented neuroteratogenic effects in ruminants; interestingly, its non-structural protein NS localizes to the nucleolus leading to nucleoplasmic translocation of NPM1 which suggests block of Pol1^46^. Similar redistribution of NPM1 was reported with the Newcastle virus which causes extensive apoptotic cell death in the brains of infected chickens^47,48^. Therefore, as in the case of ZIKV-infected neurons, one could anticipate that at least these pathogens may induce RS-mediated cytopathic effects.

However, nucleolar disruption-associated RS may not be a general feature of a host cell response to viral infection. First, some viruses activate rather than inhibit rRNA synthesis as demonstrated in human hepatoma cells that were infected with the flavivirus hepatitis C virus (HCV)^49^. Second, analysis of nucleolar proteome in cell lines that were infected with such RNA viruses as the coronavirus infectious bronchitis virus or influenza virus A revealed surprisingly small changes in nucleolar proteins unlike massive shifts of nucleolar proteomic landscape in response to the nucleolodisruptive transcriptional inhibitor Actinomycin D^50,51^. Nevertheless, focusing attention on nucleolar morphology as a correlate of RS may underestimate the occurrence of virally-induced RS. For instance, inhibition of post-transcriptional processing of rRNA due to depletion of certain ribosomal proteins or RBFs as well as increase of ribosomal biogenesis that is associated with oncogenic transformation may induce RS despite relatively normal appearance of the nucleolus^27,28^. Similar pro-RS effects may be expected in cases of viral infections that upregulate Pol1 and/or perturb rRNA processing without blocking rRNA transcription^49,52^.

Importantly, presence of RS may be determined not only by the viral species but also by differential host cell sensitivity to anti-nucleolar effects of viruses. In support of that notion, we observed greater extent of ZIKV-induced nucleolar disruption in post-mitotic neurons than in proliferating NPCs (Table 1). Moreover, overexpression of ZIKV-C was sufficient to induce RS in neurons but not hNPCs or SH-SY5Ys (Fig. 4 vs. Supplementary Fig. S9). One could speculate that such a differential sensitivity could be determined by unequal expression of RBFs that are targeted by viral proteins. As neuronal differentiation is associated with reduced Pol1 activity and downregulation of such targets for flaviviral C proteins as NPM1 or NCL^53^, post-mitotic neurons may be particularly sensitive to ZIKV-induced RS. If correct, such a scenario would predict that although ZIKV is most infectious to NPCs, at least its RS-related toxicity would be most pronounced in NPC progeny as it enters the neuronal differentiation fate. Such a differentiation stage-specific cytotoxicity could explain why the ZIKV-related microcephaly is often accompanied by symptoms of cranial collapse suggesting massive brain cell death that follows initially successful neurogenesis^54^. Future studies are needed to directly test whether neuronal differentiation-associated changes in RBF expression determine sensitivity to ZIKV-induced cell death and shape neuropathogenic cascades that underlie ZIKV-mediated damage of the developing brain.

Our data suggest that the mature capsid protein ZIKV-C is a mediator of ZIKV-induced RS and a likely contributor to neuropathogenic effects of that virus. Similar neuropathogenic mechanism may also be engaged by DENV. Nucleolar localization of ZIKV-C or DENV-C correlates with their ability to induce apoptotic RS. While there is no consensus nucleolar localization signal identified, ZIKV-C and DENV-C fit the pattern of other nucleolar proteins as they are highly basic with stretches of positively charged amino acids placed both at the N- as well as C-terminus^55,56^. Our observations indicate that as in the case of DENV-C but not JEV-C or WNV-C, the putative C-terminal RNA binding domain of ZIKV-C and/or its α4 helical domain that promotes capsid dimerization are required for nucleolar localization^22,37,57^. As the immature, membrane-anchored version of ZIKV-C had reduced ability to enter the nucleolus and induced no RS or apoptosis one could expect that blocking flaviviral protease NS3-NS2B that mediates flaviviral capsid maturation may attenuate ZIKV-induced RS^17^.

While WNV has greater neurotropic potential than ZIKV or DENV, at least in primary neuronal cultures WNV-C appeared to be relatively non-toxic as compared to ZIKV-C or DENV-C. Therefore, neurotoxicity of capsid protein alone is an unlikely determinant of a flavivirus ability to produce a neurological disease. Indeed, neurotoxicity of the capsid protein may represent one of several final effector mechanisms of flavivirus-mediated nerve cell damage. Such mechanisms could be set off at various stages of neurodevelopment and/or in distinct neural cell populations dependent on virus species/strain ability to evade antiviral response, penetrate into the developing and/or mature CNS and infect specific types of neural cells.

Recent work has shown that liquid liquid phase transition (LLPS) plays a critical role in formation of the granular component (GC) of the nucleolus^36^. LLPS is mediated by multivalent interactions of NPM1 acidic amino acids with basic amino acids of other nucleolar proteins and NPM1’s RNA binding sites with rRNA. Interestingly, that process can be disrupted by arginine-containing dipeptide repeats (DPRs) that arise due to ALS/FTLD-associated intronic expansion of the C9ORF72 gene^58^. DRPs which are believed to be key contributors to neuronal death in C9ORF72-related cases of neurodegeneration, impair dynamics of nucleolar components and compromise ribosomal biogenesis. Although DRPs are nucleolar; they also disrupt dynamics and function of other membraneless organelles such as RNA stress granules or nuclear pores. In both cases, negative impact on these structures is believed to be due to disruption of protein-protein interactions that involve basic amino acids^58^. Therefore, it is tempting to speculate that the highly basic ZIKV-C may (i) initiate similar molecular mechanism of RS as DRPs, and, (ii), like them, may have negative effects on other LLPS-dependent cellular structures including stress granules, Cajal’s bodies and/or nuclear speckles.

Interestingly, there were differences in anti-nucleolar potential of the two ZIKV strains that were tested including greater effects of the Old World strain MR766 as compared to the New World strain PRV. While it is unclear what is the basis for such differences one can speculate that contributing factors may include variation in ZIKV-C sequence (Supplementary Fig. S5) and/or strain-specific interactions between ZIKV-C and other ZIKV proteins. For instance, variants of NS1 or prM affect ZIKV ability to infect mosquito cells or induce microcephaly, respectively^59,60^.

ZIKV-C may not be the only mediator by which ZIKV induces RS. Such a possibility is supported by apparent inability of ZIKV-C overexpression to replicate moderate nucleolar disruption that is observed after ZIKV infection in hNPCs. Other flaviviral proteins including the viral RNA polymerase NS5 may also localize to nucleoli and potentially affect their function^18,49^. Anti-nucleolar effects of such alternative mediators remain to be determined by future studies.

At least in cultured cells, role of RS in mediating cytopathic effects of ZIKV is supported by recent pharmacological as well transcriptomic evidence that Tp53 contributes to ZIKV-induced apoptosis^13^. However, it is conceivable that other components of cellular ZIKV response including activation of toll-like receptor signaling, inhibition of Akt, or centrosomal/mitochondrial pathology co-operate with RS to produce ZIKV-associated brain malformations^12,35,61^. Hence, future experiments are needed to determine relative neuropathogenic contribution of RS to ZIKV-induced neurodevelopmental disruption.

Taken together, our data suggest that ZIKV-C-induced RS plays a role in ZIKV-mediated brain damage including neuronal apoptosis. Its contributions to other pathogenic consequences of ZIKV infection such as inhibition of NPC proliferation and/or self-renewal, disabling cellular anti-viral response or cytopathic effects in the placenta and/or endothelium are also possible and should be examined by future studies. Lastly, high sensitivity of neurons to anti-nucleolar effects of ZIKV suggests RS may be a common neuropathogenic mediator for various RNA viruses that target the nervous system.

## Materials and Methods

### Animals

Sprague–Dawley time-pregnant female rats were purchased from Envigo (Indianapolis, IN, USA) and on embryonic day 15, euthanized for embryo collection. All animal handling was strictly adhering to protocols that were approved by the Institutional Animal Care and Use Committee of the University of Louisville and the NIH guidelines.

### Materials

Reagents were obtained from Sigma (St. Louis, MO), VWR (Radnor, PA) or Life Technologies-Invitrogen-Gibco (Grand Island, NY) unless stated otherwise.

### Cell culture and transfections

Cultures of primary cortical rat neural progenitor cells (rNPCs) and cortical neurons were prepared from E15 rat embryos using previously described protocols with modifications^62,63^. Briefly, cerebral cortex was dissected and meninges were removed. Then, rNPCs were isolated by triturating the tissue four times with a 1-ml pipette tip and grown as neurospheres in Neurobasal medium (Gibco) supplemented with 2% B-27 Supplement (Gibco), 1 mM L-glutamine, 100 units/ml penicillin, 0.1 mg/ml streptomycin, 20 ng/ml bFGF (R&D Systems, Cat#233-FB-025), and 20 ng/ml EGF (R&D Systems, Cat#236-EG-200). Neurons were isolated by trituration of papain-digested tissue and plated onto Poly-D-Lysine- and laminin-coated 12 mm diameter plastic cover-slips that were produced in the lab from the electron microscopy-grade Mylar masks (Electron Microscopy Sciences, Hatfield). The culture medium was Basal Medium Eagle (BME, Lonza) supplemented with 10% heat-inactivated bovine calf serum (HyClone, GE Healthcare Life Sciences, Logan, UT, USA), 35 mM glucose, 1 mM L-glutamine, 100 units/ml penicillin, and 0.1 mg/ml streptomycin. The gliostatic drug cytosine arabinoside was not used due to its high toxicity in embryonic neuronal cultures. Neuronal transfections were performed on DIV2 (day *in vitro* 2) using Lipofectamine 2000 as described previously^64^. Human induced pluripotent stem cell derived neural progenitor cells (hNPCs, HIP™ Human Neural Stem Cells, MTI GlobalStem Cat# GSC-4311; a gift of Dr. Franklin D. West of the University of Georgia) were grown on Matrigel Matrix (Corning, Cat#354277)-coated dishes or cover-slips in Neurobasal medium (Gibco) supplemented with 2% B-27 Supplement (Gibco), 1 mM L-glutamine, 1x Non-Essential Amino Acids, 100 units/ml penicillin, 0.1 mg/ml streptomycin, and 50 ng/ml bFGF (R&D Systems). Passage 19–25 hNPCs were used for the experiments. Human neuroblastoma SH-SY5Y cells were maintained in DMEM medium supplemented with 10% fetal bovine serum, 100 units/ml penicillin, and 0.1 mg/ml streptomycin. A standard Lipofectamine 2000 protocol was used to transfect both hNPCs and SH-SY5Y cells as recommended by the manufacturer.

### Viruses and viral infection

ZIKV strains MR766 and PRVABC59 were provided by Dr. Barbara Johnson (CDC) and amplified in Vero76 cells as described previously^65,66^. Viral titers were determined using the virus infection center assay as described elsewhere^67^. Viral stocks were aliquoted and stored at −80 °C until use. All cells were infected at multiplicity of infection (MOI) 0.1. Infections were performed in freshly isolated rNPCs that were grown to promote neurosphere formation (5*10^5^ cells per 35 mm plate), adherent hNPCs or DIV1 rat neurons that were plated 1 day before infection; plating densities were 100,000 or 350,000 cells/well in a 24 well plate, respectively.

### Neurosphere analysis

Starting at 1 day post infection (dpi), neurospheres that were formed by rNPCs were observed and photographed daily using phase contrast microscopy (Nikon Eclipse TS100, 4x or 20x objective lenses were used for neurosphere counting or imaging, respectively). Number of neurospheres was determined in 5 randomly selected sight fields. As neurosphere forming potential differed between various preparations of rNPCs, data from independent experiments were compared after normalization to fold change of mock-infected control for each individual experiment. At 3 dpi neurosphere-grown rNPCs were dissociated with Stem Pro Accutase (Gibco) and plated onto Poly-D-Lysine/laminin-coated plastic cover-slips to obtain adherent monolayers of rNPC for microscopic analysis at a single cells level. After 16 h culture, rNPC monolayers were fixed and used for immunofluorescence staining.

### Plasmids

The following plasmids were previously described: EF1α-LacZ (EF1α promoter-driven β-galactosidase), CMV-driven DN-Tp53s (R175H^68^, V135A^69^ and Tp53-DD^70^); shLuc^71^; shL11^27^, chicken β-actin promoter-driven-EGFP-RPL4^53^; PG13 (Tp53 response element-driven luciferase reporter plasmid)^72^, EF1α-Renilla-luciferase^73^. Synthetic cDNAs encoding flaviviral proteins were custom synthesized by BioBasic (NY, USA) and cloned into mammalian expression vector pBact-16-pl^74^ downstream of the chicken β-actin promoter via HindIII-SpeI sites. These constructs included ZIKV strain BeH819966 mature capsid protein 1–104 (ZIKV-C), anchored capsid protein 1–122 (ZIKV-C/anch/), capsid deletion mutant 1–82 (ZIKV-C/1–82/), capsid deletion mutant 1–73 (ZIKV-C/1–73/), membrane glycoprotein M 1–75 (ZIKV-M), protein NS4A 1–127 (ZIKV-NS4A, GenBank genome accession: KU365779), DENV type 2 strain ‘New Guinea C’ mature capsid protein 1–101 (DENV-C, GenBank genome accession: KM204118), and WNV Type 1 A strain New York 99 mature core protein 1–105 (GenBank genome accession: HQ596519). All cloned proteins were tagged with 3xFlag tag on their N-terminal end; an additional ZIKV-C construct without any tag was also prepared. Two previously validated shRNA target sequences for rat Tp53 were cloned into pSuper shRNA expression vector (Supplementary Table S3)^39^.

### Drug Treatments

Actinomycin D (ActD) and nutlin-3 (Selleckchem #S1061, Huston, TX) were dissolved in DMSO and added to the culture media with final DMSO concentration never exceeding 0.2%. BMH-21 (a gift of Dr. Marikki Laiho, Johns Hopkins University) was dissolved in 20 mM citrate buffer (pH 6.0).

### Immunostaining

Cells were fixed in 4% formaldehyde at room temperature for 20 min followed by 15 min permeabilization in 0.5% NP-40/PBS and 1 h blocking in 5% goat serum, 0.1% Triton X-100/PBS. The following primary antibodies were used: mouse monoclonal anti-nucleophosmin-1/B23 antibody (1:750, Sigma), rat-anti Pes1 (clone 8E9, 1:200, HelmholtzZentrum Munchen, Core facility Monoklonale Antikorper, German Research Center for Environmental Health, Munich, Germany), humanized 4G2 monoclonal anti-Flavi-E (1:500, a gift from Dr. Nobuyuki Matoba, University of Louisville), rabbit anti-β-galactosidase (1:1000, MP), and rabbit anti-GFP (1:1000, MBL). The following secondary antibodies were used Alexa Fluor 488 goat anti-mouse IgG, Alexa Fluor 488 goat anti-rat IgG, Alexa Fluor 488 goat anti-rabbit IgG, Alexa Fluor 594 anti-human IgG, and Alexa Fluor 594 anti-rabbit IgG (all Invitrogen, in all cases dilution was 1:300).

### Flow cytometry analysis of hNPC infection

HIP-hNPCs cultured for 18 h in a Matrigel-coated 24-well plate were infected by incubating with diluted virus in a volume of 0.5 mL (MOI 0.5). The next day the media was removed and replenished with fresh media (Day 1). Cells were dissociated with Accutase followed by two times of washing with HBSS by centrifugation at a low speed. Cells were fixed in 4% paraformaldehyde in PBS for 20 min. at 4 °C and blocked and permeabilized in a blocking buffer (PBS with 1% BSA and 0.05% sapponine) for overnight. The cells were stained with 4G2 mouse monoclonal anti-E (1 µg/mL) (CellSignal D3E9, 1:200) at 4 °C for one hour that was followed by Alexa Fluor 488 goat anti-mouse IgG (Jackson ImmunoResearch 1.5 µg/mL). FACS analysis was done with BD LSRFortessa and FlowJo software.

### *In Situ* Run-On Assay

Neuronal nascent RNA was labeled with the RNA precursor 1 mM 5-ethynyl uridine (5-EU; Berry &Associates, Inc., Dexter, MI, dissolved in water) for 2 h which was detected using the Click-It reagent Oregon Green® azide (Invitrogen) as previously described^71^.

### Quantification of the fluorescence intensity

Immunofluorescently-labeled cells were visualized with Zeiss Observer.Z1 fluorescent microscope using 40x or 63x lenses. Digital pictures were captured using Zeiss AxioVision and converted to gray scale TIFF files. Identical exposure times were used for all pictures that were used for comparative analyses. Fluorescence intensity was quantified using “Integrated Density” parameter in the ImageJ software with background correction. Whenever territory of the nucleolus was measured an “Area” parameter was used. Fluorescence intensity was converted to fold control that was defined by a ratio of an individual value to the average value of the control group as indicated for each set of experiments.

### Quantification of apoptosis

To detect apoptosis in infected or transfected neurons cells were immunostained for Flavi-E or the transfection marker β-gal and counter-stained with 2.5 µg/mL Hoechst-33258. Cells with condensed or fragmented nuclear chromatin were scored as apoptotic. At least 150 infected and/or transfected cells were analyzed for each condition in each experiment.

### Cell survival assay

Cell survival assay using luminometric ATP level measurements was performed in ZIKV-infected hNPCs as previously described^75^.

### Transcription assay

Dual luciferase reporter assay was performed after co-transfection of the p53-driven-Firefly luciferase construct PG13 and the EF1α-Renilla-luciferase plasmid. Luciferase activities were measured using commercial kits (Promega) and the Berthold Orion II luminometer as previously described^76^. Transcriptional activity was expressed as a Renilla-normalized luciferase activity.

### Statistical analysis

Single cell level data (such as fluorescence intensity) where n > 10/group and sufficiently powered normality testing was possible were analyzed by one-way ANOVA and Tukey or Fisher *post-hoc* tests as indicated; all other data were analyzed using non-parametric tests (Kruskall-Wallis ANOVA for comparing multiple groups or Mann-Whitney *u*-test for pairwise comparisons).

### Data availability statement

The datasets generated during the current study are available from the corresponding author on reasonable request.

## Electronic supplementary material

Supplementary Information
