# Supplementary material for: Ribosomal stress and Tp53-mediated neuronal apoptosis in response to capsid protein of the Zika virus
Source: Sci Rep. 2017 Nov 30;7:16652. doi: 10.1038/s41598-017-16952-8 (PMC5709411; doi:10.1038/s41598-017-16952-8)
Supplement: Supplementary file 1 — Supplementary Information [file 41598_2017_16952_MOESM1_ESM.pdf]

Supplementary Information

Ribosomal stress and Tp53-mediated neuronal apoptosis in response to capsid protein of the Zika virus.

Lukasz P. Slomnicki, Dong-Hoon Chung, Austin Parker, Taylor Hermann, Nolan L. Boyd, and, Michal Hetman

Supplementary Table S1. Cell type is a significant factor affecting severity of ZIKV-induced RS as revealed by combined two-way ANOVA analysis of NPM1 immunofluorescence data from ZIKV infected rNPCs, hNPCs and rat embryonic cortical neurons.

**FACTOR**	**PARAMETERS OF RS (nucleolar disruption)**		
	**Nucleolar NPM1 (FI ratio of nucleolar to nuclear signal, mock-normalized)**	**Nucleolar NPM1 (territory, mock-normalized)**	**Number of NPM1-positive nucleoli/cell (mock-normalized)**
**Virus Infection (mock or MR766 or PRV)**	$F_{2/509}$=165.246, $p<0.001$	$F_{2/509}$=104.414, $p<0.001$	$F_{2/510}$= 12.253, $p<0.001$
**Cell Type (rNPC, hNPC or rat neurons)**	$F_{2/509}$=4.204, $p<0.05$	$F_{2/509}$= 11.166, $p<0.001$	$F_{2/510}$= 3.211 $p<0.05$
**Virus Infection X Cell Type**	$F_{4/509}$=2.753, $p<0.05$	$F_{4/509}$= 3.857, $p<0.01$	$F_{4/510}$= 2.774, $p<0.05$

Supplementary Table S2. Effects of ZIKV-C, DENV-C and WNV-C on accumulation of 5EU-labelled nascent RNA in nucleoli and nuclei of primary rat cortical neurons.

Transfected plasmid (number of cells analyzed)	Nucleolar signal (fluorescence intensity normalized to total nuclear signal, fold EV control ±SEM)	Total nuclear signal (fluorescence intensity, fold EV control ±SEM)
Empty Vector /EV/ (n=43)	1±0.072	1±0.073
FL-ZIKV-C (n=40)	0.73±0.063 [a]	0.55±0.038 [a, c]
FL-DENV-C (n=38)	0.75±0.062 [a]	0.69±0.074 [a, c]
FL-WNV-C (n=40)	1.14±0.053 [b]	0.75±0.045 [a, c]

[a] $p<0.001$, as compared to EV (*u*-test); [b] $p>0.05$, as compared to EV (*u*-test); [c] reduction of nascent RNA signal in whole nuclei suggests inhibition of extranucleolar transcription and/or lower uptake of 5EU into cells; hence, effects of ZIKV-C or DENV-C on nucleolar transcription may be potentially underestimated.

Supplementary Table S3. Oligonucleotide sequences that were used to generate shRNAs against rat Tp53 (NM_030989).

shTp53	strand	oligonucleotide sequence (Tp53 target sequences are underlined) [a]
#1	sense	5’gatccccgtcagggacagccaagtctttcaagagaagacttggctgtccctgactttttta3’
	antisense	5’agcttaaaaagtcagggacagccaagtcttctcttgaaagacttggctgtccctgacggg3’
#2	sense	5’gatccccgtgccatggccatctacaattcaagagattgtagatggccatggcactttttta3’
	antisense	5’agcttaaaaagtgccatggccatctacaatctcttgaattgtagatggccatggcacggg3’

[a] the shRNAs target sequences were previously validated (PLOS One, 2014, 9: e108257). After annealing, oligonucleotides were subcloned into a pSuper vector (OligoEngine) digested with BglII and HindIII.

Supplementary Figure S1. Compromised neurosphere formation in ZIKV-infected rat embryonic neuroprogenitor cells (rNPCs). Freshly isolated rNPCs were infected with ZIKV strains MR766 or PRVABC59 at MOI 0.1 and grown as neurospheres for up to 3 days post infection (dpi). In D-F, at 3 dpi cells were dispersed, and cultured as a monolayer for 16 h to enable microscopic analysis at a single cell level. **(a)** Representative phase contrast micrographs from ZIKV-infected rNPCs cultures at 3 dpi. Arrows point neurospheres in mock-infected cultures. Neurosphere growth was disrupted by either strain of ZIKV as rNPCs attached to the plates. **(b)** ZIKV effects on neurosphere number over time after infection. Data from a representative experiment are shown; similar results were obtained in an independent experiment. **(c)** At 3 dpi, significant reduction of neurosphere number was observed; data represent averages of 3 independent experiments. **(d)** Flavi-E immunofluorescence confirmed ZIKV infection (% Flavi-E-positive cells±SEM is indicated on the images). **(e-f)** ZIKV strain MR766 increased apoptosis of infected (*i.e.* Flavi-E-positive) rNPCs. An arrowhead in **(e)** indicates a Flavi-E-stained cell with apoptotic condensation and fragmentation of nuclear chromatin as revealed by DNA counterstaining with Hoechst-33258. Data in **(f)** represent 4 sister cultures from 2 independent experiments. Data were analyzed by *u*-test NS, $p>0.05$; *, $p<0.05$.

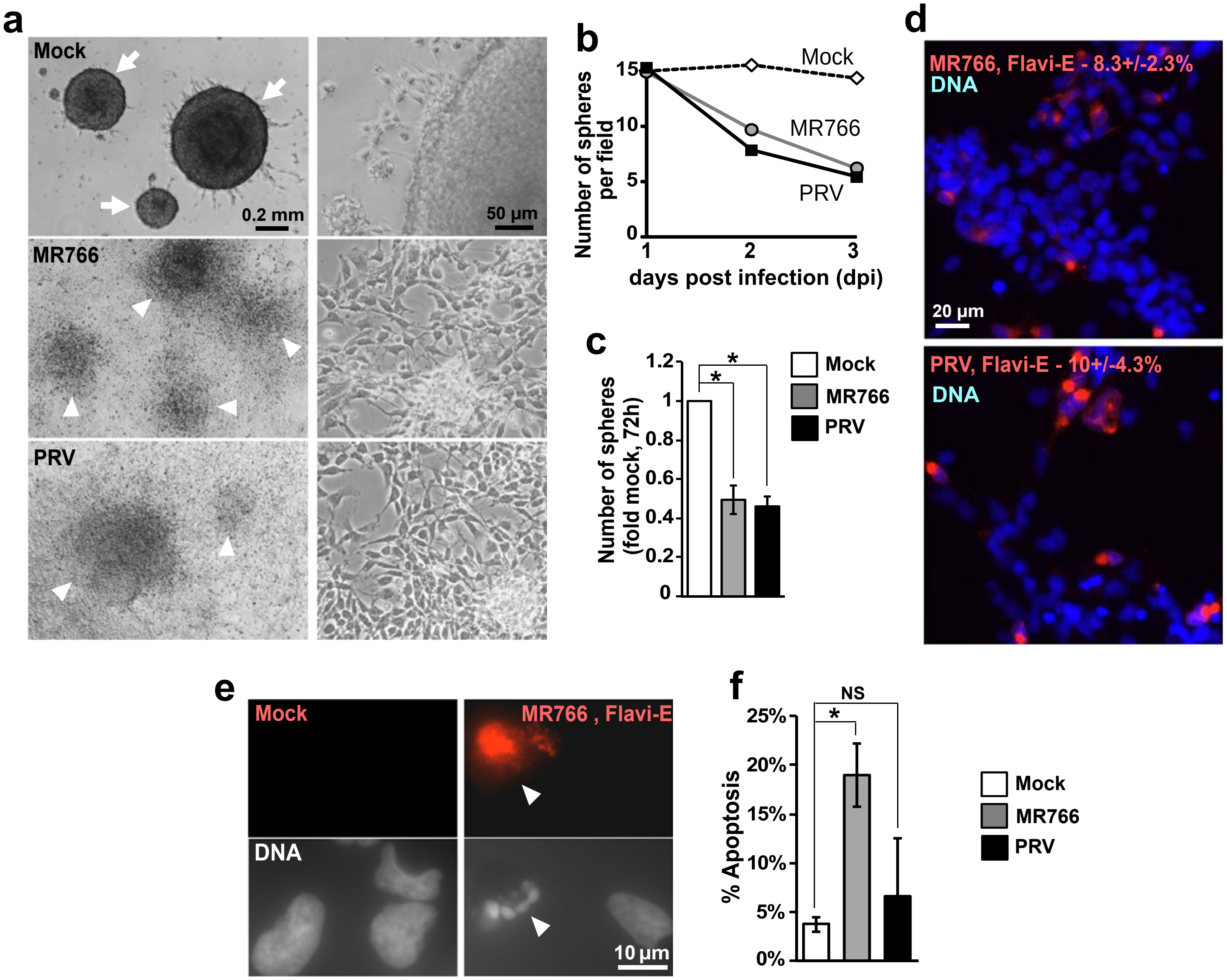

Slomnicki et al. Supplementary Fig. S1

Supplementary Figure S2. Anti-survival effects of ZIKV in human iPSC-derived neuroprogenitor cells (hNPCs). Monolayer-grown hNPCs were infected with ZIKV strains MR766 or PRVABC59 (PRV) at MOI 0.1. **(a)** ZIKV infection had negative effects on hNPC viability. Note faster cell death in MR766-infected cultures; data are averages of 6 sister cultures from a representative experiment. **(b)** Flow cytometry analysis of Flavi-E-immunostained hNPCs reveals extent of ZIKV infection. Due to low number of cells, no reliable data were obtained for MR766 beyond 2 dpi; data are averages of 2 sister cultures from a representative experiment. **(c)** Representative images depicting non-apoptotic ZIKV-infected hNPCs (*i.e.* Flavi-E-positive) that were co-immunostained for the nucleolar marker NPM1 at dpi 1; DNA was counterstained with Hoechst-33258. Nuclear contours of these cells are marked by dotted lines. As hNPC survival is not compromised at dpi 1, reduced fluorescence intensity (FI) of NPM1 precedes ZIKV-mediated cell death.

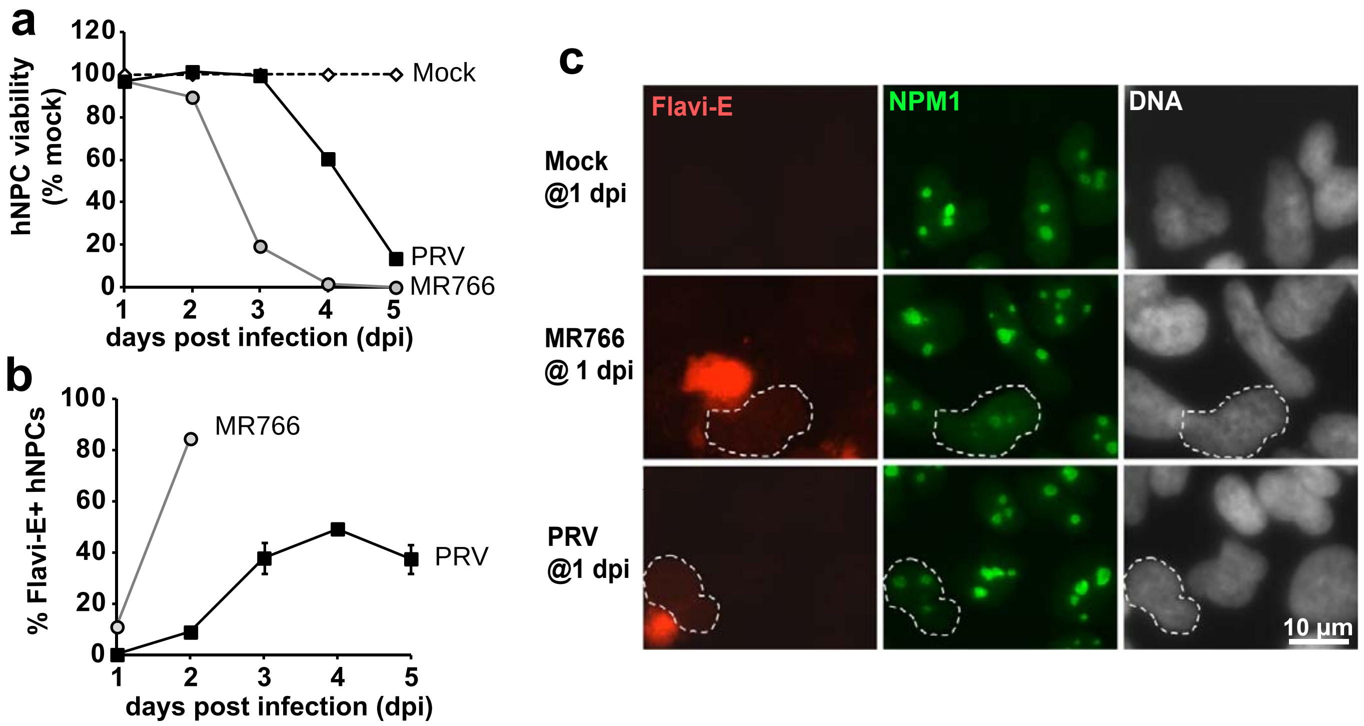

Slomnicki et al. Supplementary Fig. S2

Supplementary Figure S3. Anti-nucleolar effects of ZIKV MR766 in hNPCs at 4 dpi. Human NPCs were infected with MR766 ZIKV and their nucleolar integrity was analyzed by Flavi-E/NPM1 co-immunostaining at 4 dpi as described for Fig. 2. Although most cells were lost at that time, 85.4%±4.73% of those that survived were ZIKV-infected (*i.e.* Flavi-E-positive). **(a)** Nucleolar NPM1 signal intensity was reduced in Flavi-E-positive hNPC. **(b)** NPM1-positive nucleolar territory also decreased. **(c)** Number of NPM1-positive nucleoli did not change. Data represent at least 53 randomly selected individual cells from two independent experiments; NS, $p>0.05$; *, $p<0.05$; ***, $p<0.001$ (one-way ANOVA and Tukey's *post-hoc* tests).

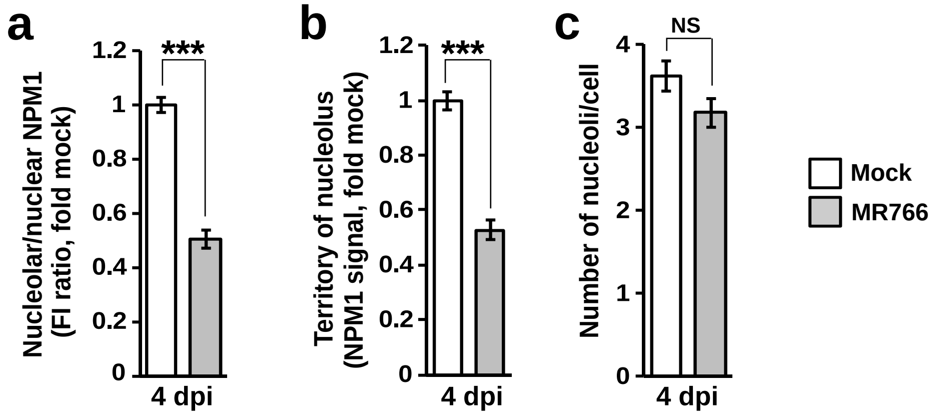

Slomnicki et al. Supplementary Fig. S3

Supplementary Figure S4. Disruption of nucleolar PES1 staining in ZIKV-infected hNPCs. Human NPCs were infected with ZIKV and their nucleolar integrity was analyzed by Flavi-E/PES1 co-immunostaining at 1 dpi as described for Fig. 2; only cells with uncondensed nuclear chromatin were selected for the analysis. **(a)** Representative images of PES1 staining in ZIKV- (*i.e.* Flavi-E-positive) neurons. Nuclear contours depicting Hoechst-33258-stained nuclear chromatin of infected cells are outlined with dotted lines. Nucleolar PES1 signal intensity **(b)** as well as territory **(c)** was reduced in hNPC that were infected with either strain of ZIKV; however, both parameters were affected more profoundly by MR766 than PRVABC_59 (PRV vs. MR766 $p<0.001$ or $p<0.05$ for signal intensity or territory, respectively; Tukey's test). **(d)** MR766 also reduced number of nucleoli per cell. Data represent at least 37 randomly selected individual cells from two independent experiments; NS, $p>0.05$; *, $p<0.05$; **, $p<0.01$; ***, $p<0.001$ (one-way ANOVA and Tukey's *post-hoc* tests).

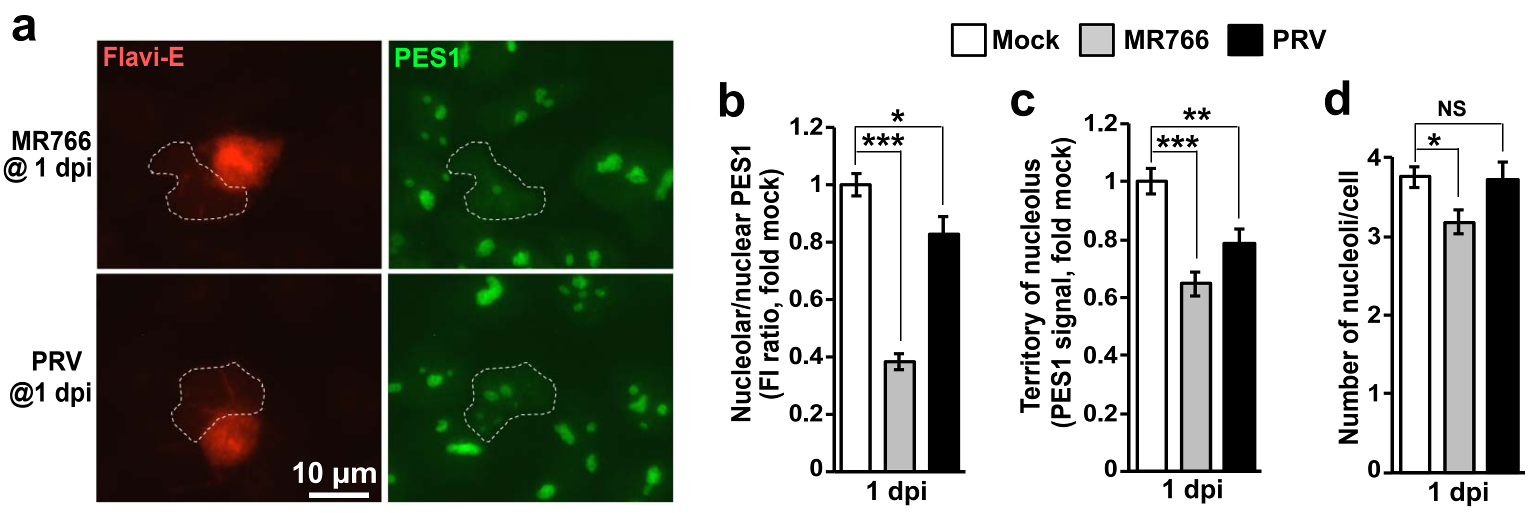

Slomnicki et al. Supplementary Fig. S4

Supplementary Figure S5. Divergence of capsid protein amino acid sequence between various strains of ZIKV (a) or ZIKV-related flaviviruses (b). (a) Multialignemt of amino acid sequences of ZIKV-C from different Zika virus strains including MR-766 (GenBank Genome Accession: KX377335), PRVABC-59 (KX377337), BeH819966 (Brazil, KU365779; this sequence was used to prepare synthetic ZIKV constructs that were used in the current work), and, three ZIKV isolates from microcephaly cases: BeH823339 (KU729217, Science 352 (6283):345-349, PubMed PMID: 27013429), Natal RGN (KU527068, N. Engl. J. Med. 374 (10), 951-958, PubMed PMID: 26862926), Brazil-ZKV2015 (Brazil, KU497555, Lancet Infect Dis. 16(6):653-660, PubMed PMID: 26897108.). Sequences were compared using the MultAlin multiple sequence alignment software (Nucl Acids Res., 16 (22), 10881-10890, PubMed PMID: 2849754). **(b)** Multialignemt of amino acid sequences of the capsid protein C of: West Nile virus type 1A, New York 99 strain (GenBank Genome Accession: HQ596519), Brazilian Zika virus strain BeH823339 (KU729217), and Dengue virus type 2-AsianII strain (New Guinea C; KM204118). The sequences were compared using the PRALINE multiple sequence alignment software with the conservation index (consistency) tool (Comput Chem. ,15;23(3-4):341-64, PubMed PMID: 10404624).

a

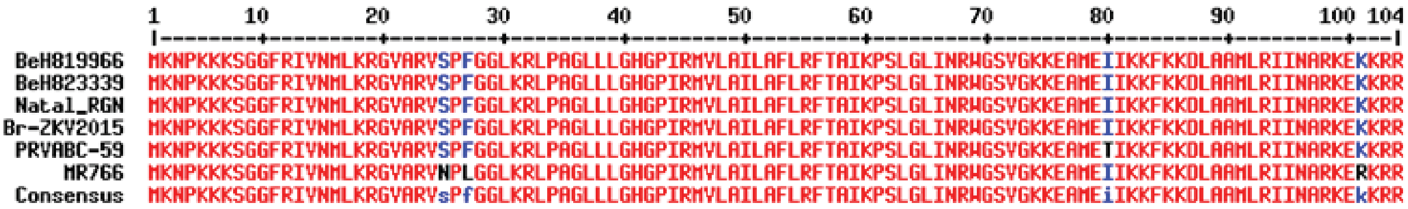

b

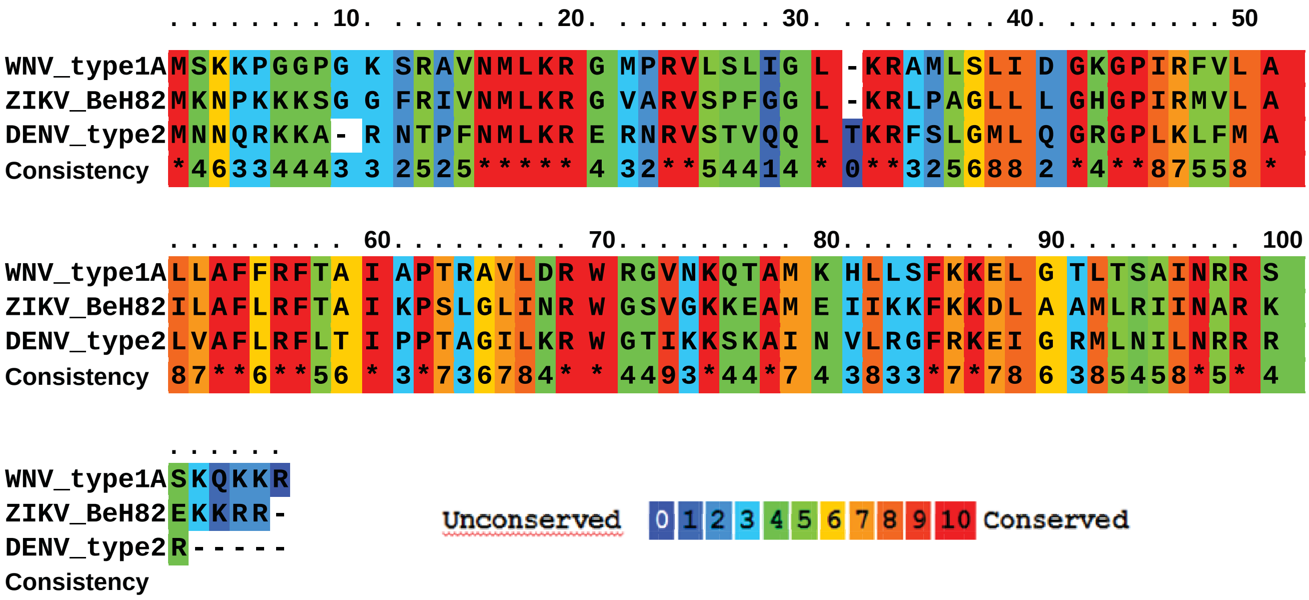

Slomnicki et al. Supplementary Fig. S5

Supplementary Figure S6. Representative images of subcellular localization of the overexpressed FL-ZIKV-C. Neurons were transfected and analyzed as for Fig. 3a (fixation at 48 h post transfection, co-immunostaining for FL-ZIKV-C and NPM1). Cells in the top two rows have prominent nucleolar enrichment of ZIKV-C and show strong NPM1 staining in the nucleolus. The two middle rows depict cells with weaker nucleolar enrichment of ZIKV-C which may be related to nucleolar stress as indicated by weaker nucleolar NMP1 and NMP1 diffusion throughout nucleoplasm (arrows). The two bottom rows show cells with no obvious nucleolar enrichment of ZIKV-C despite maintained nucleolar NPM1 (such cells were counted as having no nucleolar ZIKV-C in Fig. 3b); in one of these cells, nuclear structures that weakly stained for Fl-ZIKV-C (arrowheads) did not overlap with NMP1-positive nucleoli.

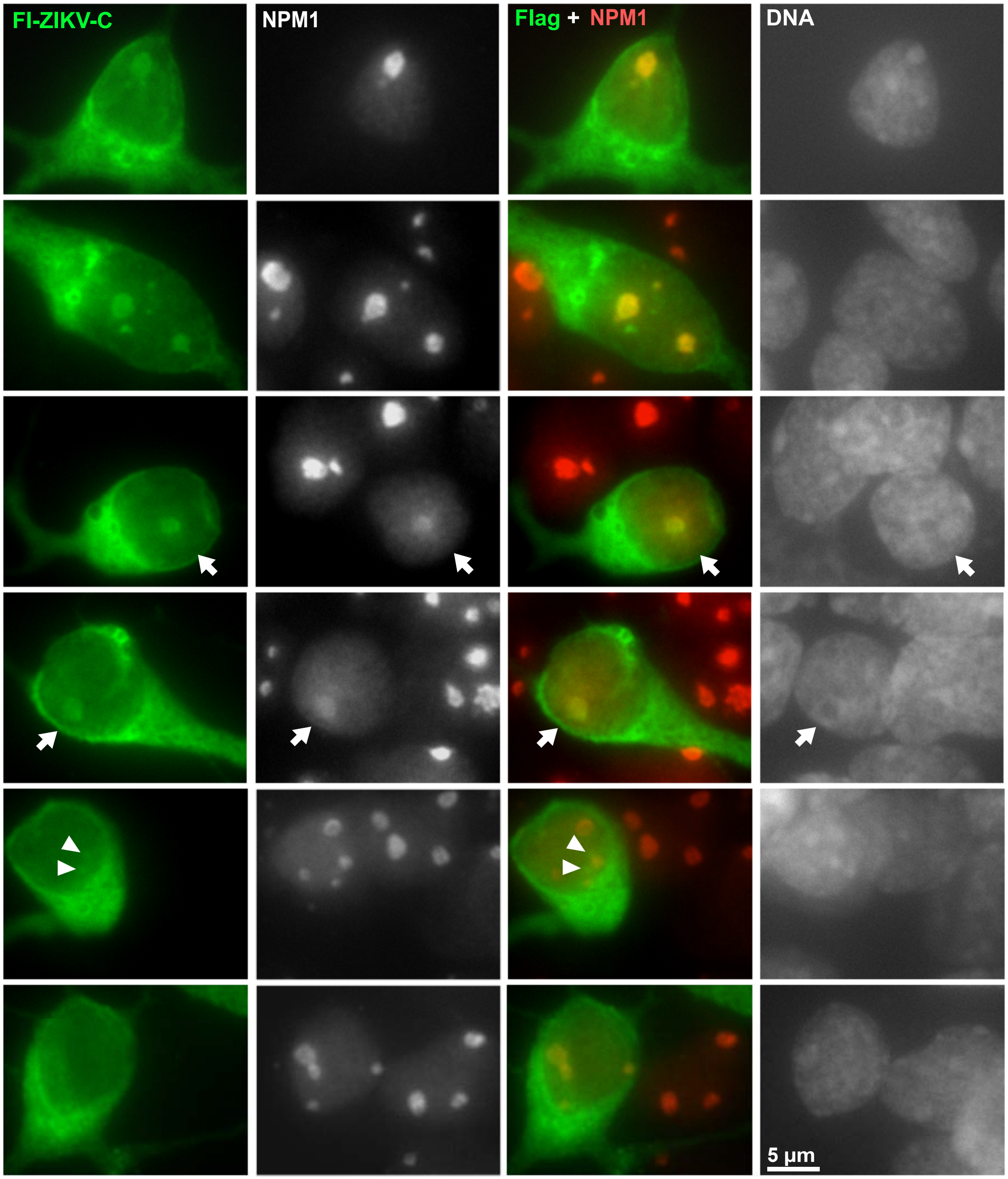

Slomnicki et al. Supplementary Fig S6

Supplementary Figure S7. Representative images of NPM1 staining in empty vector (EV)- or Fl-ZIKV-C-transfected neurons. Neurons were transfected and analyzed as for Fig. 4a (fixation at 48 h post transfection, co-immunostaining for the transfection marker β-gal and NPM1). Note that nucleolar stress is present in ZIKV-C- (arrows) but not EV-transfected neurons (arrowheads) as indicated by the weaker nucleolar NMP1 signal and increased nucleoplasmic NMP1. Grayscale-converted NPM1 images with increased brightness are also shown to visualize nucleolar NPM1 signal in a subpopulation of cells with low level expression of NPM1. Regardless of NMP1 expression level, no signs of nucleolar stress are present in non-transfected (*i.e.* β-gal-negative) cells. Note that heterogeneity of NPM1 expression did not have a major impact on evaluation of nucleolar integrity (such as that in Fig. 4b-h) as (*i*) cells with very low NPM1 expression were rarely transfected (<10% of the transfected population), (*ii*) random cells were selected for quantitative image analysis, (*iii*) nucleoplasmic diffusion of NMP1 was a prominent feature of nucleolar disruption in ZIKV-infected primary neuronal cultures; such phenomenon would have been detected irrespective of the initial size of the nucleolus, and, (*iv*) relative nucleolar NPM1 fluorescence intensity content was normalized to the NPM1 signal from the whole nucleus.

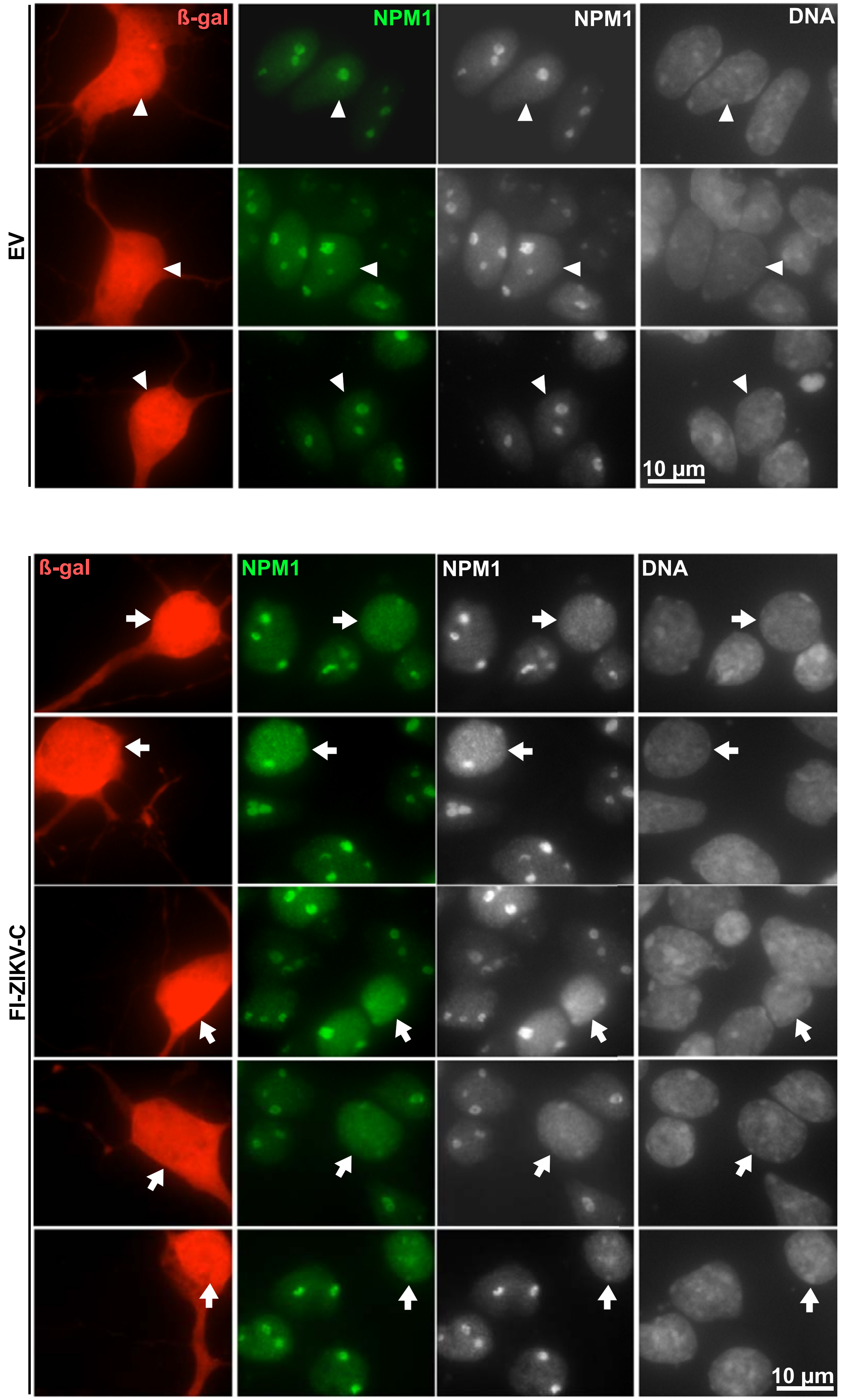

Slomnicki et al. Supplementary Fig. S7

Supplementary Figure S8. ShRNAs targeting rat Tp53 reduced activity of the Tp53-driven luciferase reporter construct. Neurons were transfected and analyzed as for Fig. 5d except shRNA constructs were added as indicated at 0.3 μg/3.5x10^5 cells; an shRNA targeting GFP was used as a control. After 24 h, cells were treated with 5 μM nutlin or its vehicle (0.2% DMSO) for 8 h followed by cell lysis and luciferase activity assay. ShRNA against Tp53 reduced basal activity of the Tp53 reporter by at least 60%. Similar reductions were also observed in nutlin-treated neurons which by itself produced moderate activation of the reporter (1.46 fold of vehicle treated control in shGFP-transfected neurons). Data represent six sister cultures from two independent experiments; NS, $p>0.05$; *, $p<0.05$; **, $p<0.01$ (*u*-test).

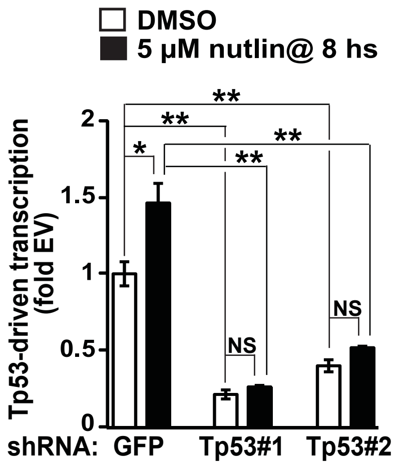

Slomnicki et al. Supplementary Fig. S8

Supplementary Figure S9. Overexpression of ZIKV-C does not induce nucleolar stress in human iPSC-derived NPCs or the human neuroblastoma cell line SH-SY5Y. (a-g) Cells were transfected with expression plasmids for a transfection marker (β-gal or RFP) and ZIKV-C (150 + 150 ng plasmid DNA/well in a 24-well plate which was seeded 1-2 days before transfection with $5x10^4$ cells/well); empty vector (EV) was used as a control for ZIKV-C. At 48 h post transfection, some cells were fixed and stained for NMP1 and β-gal **(a-d)** while others were used for the *in situ* run on assay **(e)** as described for Fig. 4i-j. Note limited effects of ZIKV-C on NMP1-positive nucleoli including 23% reduction of nucleolar territory **(b)** of hNPCs and lower number of nucleoli/cell in SH-SY5Y cells **(d)**. Other parameters such as nucleoplasm-normalized nucleolar NPM1 fluorescence intensity (FI) **(b, d)** or nucleolar accumulation of nascent RNA were unaffected **(f, g)**. (**h-i)** Cells were transfected as for Fig. 5d and ZIKV-C effects on transcriptional activity of Tp53 were evaluated using the Tp53-driven luciferase reporter plasmid. ZIKV-C did not stimulate Tp53 activity. Conversely, Tp53 was activated by 8 h treatment with the MDM2/HDM2 inhibitor nutlin that stabilizes Tp53. Thus, unlike in neurons (Fig. 4-5), overexpression of ZIKV-C in hNPCs or SH-SY5Y cells is insufficient to activate the RS-Tp53 pathway. Data from two independent experiments are shown; numbers of individual cells that were analyzed are at least 51 in **(b)**, 44 in **(d)**, 55 in **(f)**, and, 43 in **(g)**; in **(h-i)**, averages of 6 sister cultures are depicted; NS, p>0.05; *, p<0.05; **, p<0.01; ***, p<0.001 (one-way ANOVA in **(b, d, f-g)** and *u*-test in **(h-i)**).

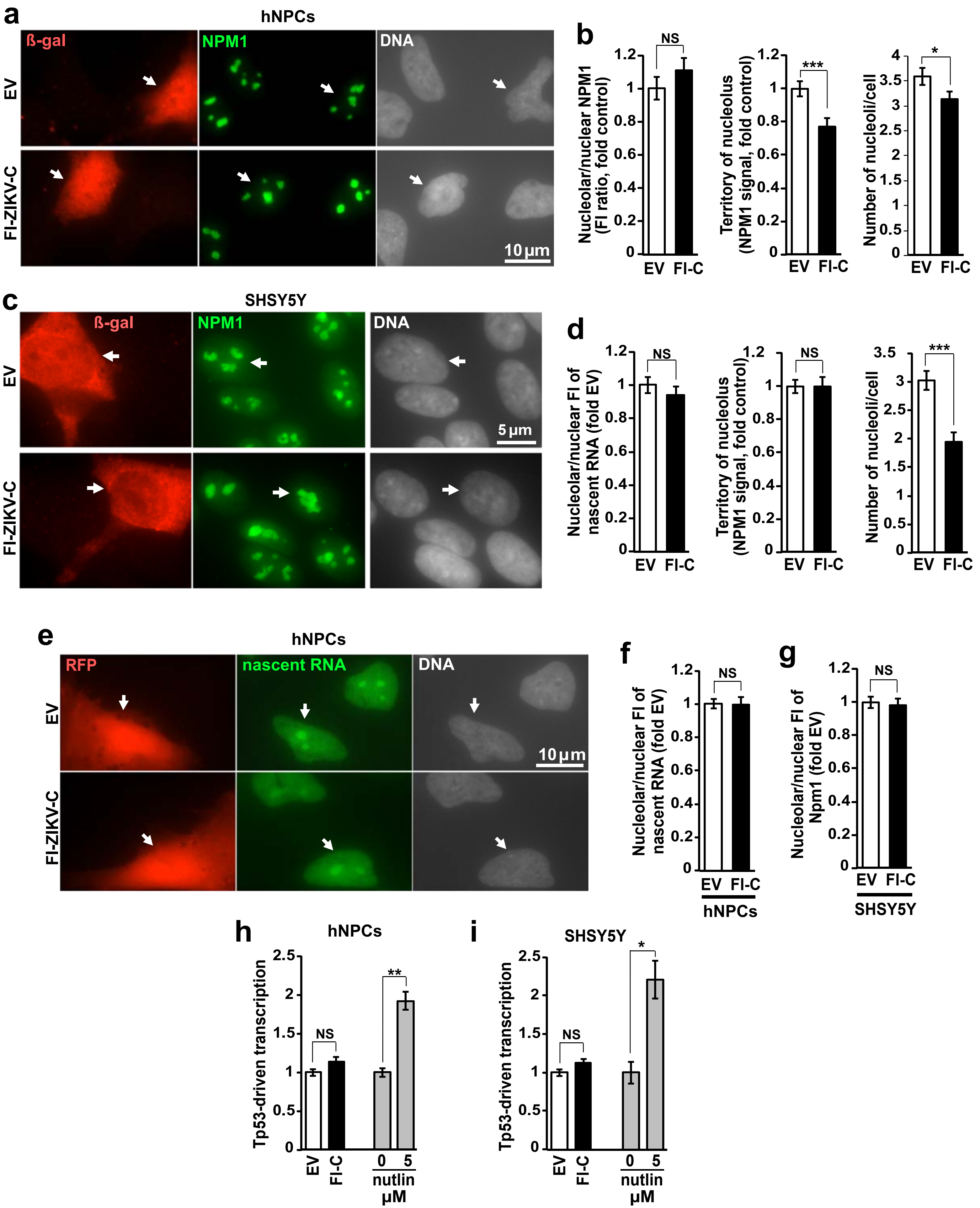

Slomnicki et al. Supplementary Fig. S9

Supplementary Figure S10. Greater nucleolar enrichment of DENV-C than WNV-C. Rat embryonic cortical neurons were transfected with Flag(Fl)-DENV-C or Fl-WNV-C and immunostained for Flag and NPM1 as described for Fig. 6. The ImageJ "Plot profile" algorithm was used to determine pixel intensity along the indicated lines that were drawn to intersect the centers of the nucleolus-like granules that stained positive for Fl. The analyzed representative images are from Fig. 6a. Note the overlap of the signals for Fl-DENV-C and NPM1 **(a-b)**. While overlap is also present for Fl-WNV-C **(c-d)**, nucleolar peaks of WNV-C are smaller than those for FL-DENV-C indicating greater nucleolar enrichment of the latter construct.

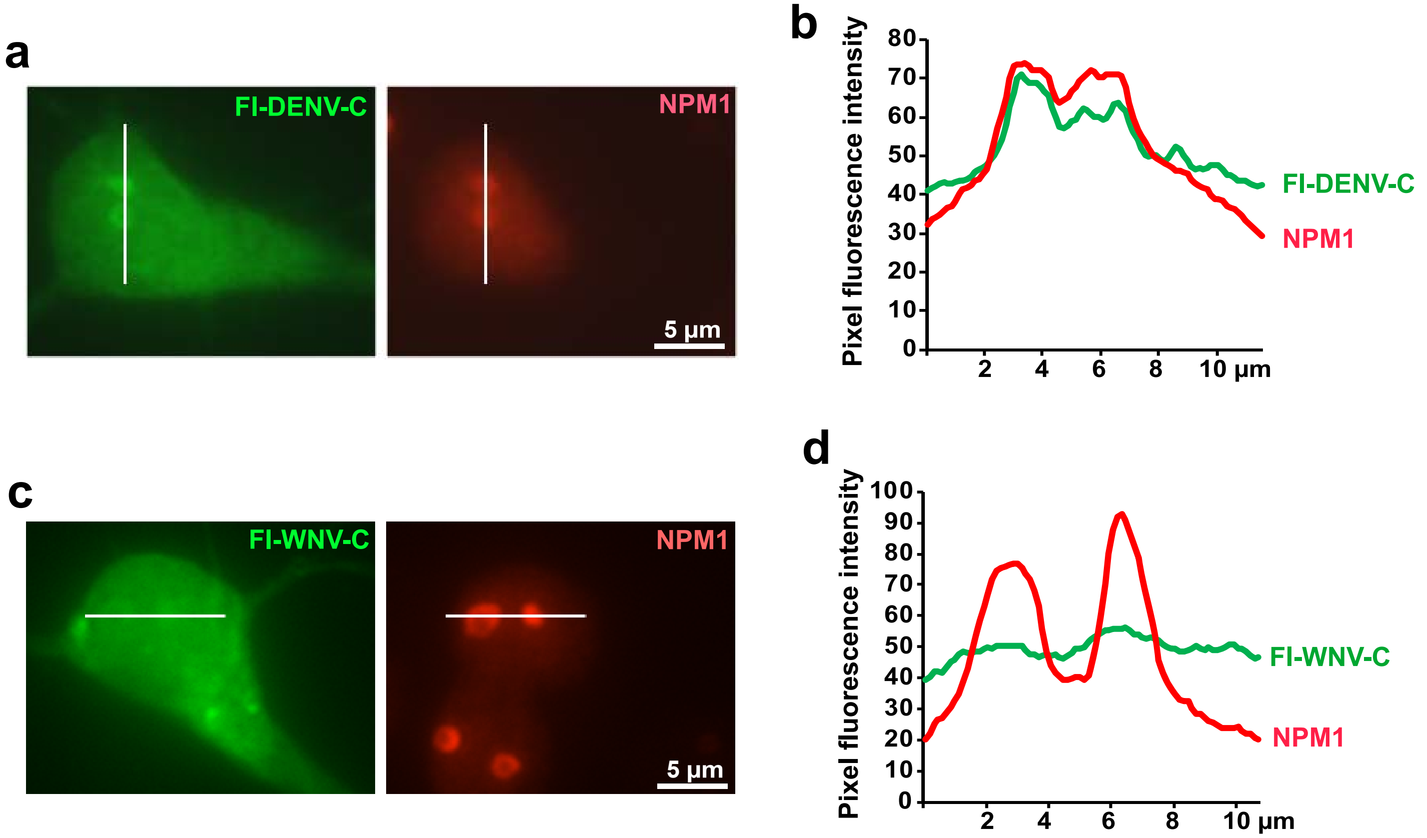

Slomnicki et al. Fig. Supplementary S10
